# Neuron-specific expression of murine thyroid hormone transporters Mct8 and Oatp1c1 is dispensable for hippocampus-dependent neuronal functions

**DOI:** 10.3389/fendo.2026.1781214

**Published:** 2026-03-17

**Authors:** Andrea Alcaide-Martin, Rim Jaber, Anita Boelen, Heike Heuer, Steffen Mayerl

**Affiliations:** 1 Department of Endocrinology, Diabetes and Metabolism and Center for Translational Neuro- and Behavioral Sciences (C-TNBS), University Hospital Essen, University Duisburg-Essen, Essen, Germany; 2 Endocrine Laboratory, Department of Laboratory Medicine, Amsterdam University Medical Center (UMC), University of Amsterdam, Amsterdam, Netherlands; 3 Research Institute Amsterdam Gastroenterology Endocrinology & Metabolism (AGEM), Amsterdam University Medical Center (UMC), Amsterdam, Netherlands

**Keywords:** GABAergic interneurons, glutamatergic neurons, Mct8, Oatp1c1, thyroid hormones

## Abstract

**Introduction:**

Global absence of the thyroid hormone (TH) transporters monocarboxylate transporter 8 (Mct8) and organic anion transporting polypeptide 1c1 (Oatp1c1) in Mct8/Oatp1c1 double knockout (M/O dKO) mice results in a severe central TH deficit due to impaired TH transport across brain barriers. This deficit is accompanied by pronounced abnormalities in inhibitory and excitatory neuronal systems in the brain. However, it remains unclear whether these alterations arise solely from central TH deficiency or whether Mct8 and Oatp1c1 also exert cell-autonomous functions in neurons.

**Methods:**

Immunofluorescence and fluorescent in situ hybridization (FISH) were used to first characterize the expression of Mct8 and Oatp1c1 in GABAergic interneuron subpopulations. Two conditional mouse lines with deletion of both transporters either in all GABAergic (GABA del mice) or glutamatergic neurons (Glut del mice) were then generated. Serum TH concentrations and TH-dependent gene expression were assessed by LC–MS/MS and FISH, respectively. Using immunofluorescence and qPCR, components of the GABAergic and glutamatergic systems as well as adult hippocampal neurogenesis were evaluated. Functional analyses were performed including pilocarpine-induced seizure susceptibility, novel object recognition and elevated plus maze tests.

**Results:**

Serum TH concentrations and TH-regulated gene expression in the brain were unaltered in both conditional mouse lines. No differences in GABAergic markers and expression of glutamatergic ionotropic receptor subunits were found in the hippocampus of GABA del and Glut del mice alongside a normal seizure susceptibility and an unaltered neurogenic program in the adult dentate gyrus. Finally, evaluation of hippocampus-dependent behaviors did not reveal alterations upon neuronal Mct8/Oatp1c1 deficiency, whereas M/O dKO mice exhibit abnormal anxiety-related behavior.

**Conclusions:**

Together, these data point to the central hypothyroid state of M/O dKO mice as the main cause of the neuronal alterations present in these animals and rule out a major cell-autonomous function of Mct8/Oatp1c1 in GABAergic or glutamatergic neurons.

## Introduction

Thyroid hormones (TH), 3, 3’, 5’-triiodothyronine (T3) and 3, 3’, 5, 5’-tetraiodothyronine (thyroxine, T4), are crucial for proper brain development and function, as they coordinate a wide range of neurodevelopmental processes (1, 2). TH exert their effects primarily through binding to nuclear TH receptors (TRs), ligand-dependent transcription factors that modulate the expression of TH-regulated genes (3) and their uptake into target cells across cell membranes is regulated by specialized TH transporters. The monocarboxylate transporter 8 (MCT8), encoded by the *SLC16A2* gene, is to date the most specific TH transporter known and exhibits a widespread expression in peripheral tissues and the brain (4–11). Inactivating mutations in MCT8 cause a severe psychomotor retardation in humans also known as Allan-Herndon-Dudley syndrome (AHDS), characterized by elevated serum T3 and reduced serum T4 concentrations (12–14). Moreover, these patients present neurological abnormalities such as alterations in the development of the cerebro-cortical GABAergic system and epileptic seizures (15–17). These observations underscore the essential role of MCT8 in brain TH economy required for proper brain development.

Because of interspecies differences, inactivation of Mct8 alone is insufficient to induce severe neurological impairments in mice, and only the combined loss of Mct8 and the organic anion transporting polypeptide 1c1 (Oatp1c1, encoded by the *Slco1c1* gene) recapitulates alterations similar to MCT8 deficiency (18–21). Mct8/Oatp1c1 double knockout (M/O dKO) mice exhibit markedly impaired TH transport into the brain and, consequently, severely reduced central T3 and T4 concentrations. In addition, M/O dKO animals present alterations in the development of GABAergic inhibitory interneurons in both the cerebral cortex and hippocampus comparable to MCT8 patients (18, 22). Recent analyses further demonstrated signs of abnormal glutamatergic excitatory neurotransmission and brain excitability in M/O dKO animals, as evidenced by their increased susceptibility to pilocarpine-induced status epilepticus (22). Collectively, these findings suggest that M/O dKO mice undergo abnormal neuronal development.

It remains unclear whether the neuronal alterations observed in M/O dKO mice arise exclusively from impaired TH transport across brain barriers or whether Mct8 and Oatp1c1 also exert an additional cell-autonomous function in regulating TH uptake into neurons. Discriminating between these mechanisms is important, as it will guide the development of more targeted therapeutic strategies for MCT8 deficiency.

In this study, we investigated potential neuron-specific roles of Mct8/Oatp1c1 by generating conditional mouse models with cell-specific deletion of both TH transporters in either all GABAergic inhibitory or glutamatergic excitatory neurons. TH homeostasis in both conditional knockout animal lines appeared normal. Furthermore, neuronal development, GABAergic and glutamatergic marker expression, and hippocampus-dependent functions were unaltered. In contrast, behavioral abnormalities were present only in M/O dKO mice. These data argue against the hypothesis of a major neuron-autonomous function of Mct8/Oatp1c1 in mice.

## Materials and methods

### Animals

All animal studies were executed in accordance with the European Union (EU) directive 2010/63/EU on the protection of animals used for scientific purposes and in compliance with local regulations by the Animal Welfare Committee of the Landesamt für Verbraucherschutz und Ernährung Nordrhein-Westfalen (LAVE; Recklinghausen, Germany; approval codes AZ81-02.04.2021.A178 and AZ81-02.04.2022.A156). All animals were housed in IVC cages and kept at constant temperature (22°C) and light cycle (12 h light, 12 h dark). Animals were provided with standard laboratory chow and tap water *ad libitum*.

All animals used in this study were on a C57BL/6 background. Mct8 fl/fl and Oatp1c1 fl/fl (Mct8/Oatp1c1 double floxed (dfl)) mice, Mct8 knockout (Mct8-KO) and Mct8/Oatp1c1 double knockout (M/O dKO) mice were generated, interbred and genotyped as explained elsewhere (18, 21, 23, 24). To abolish Mct8/Oatp1c1 expression in GABAergic neurons, Mct8/Oatp1c1 dfl mice were mated with transgenic animals expressing a constitutively active Cre-recombinase under the glutamic acid decarboxylase 2 promoter (*Gad2*) (Gad2tm2(cre)Zjh/J, Jax stock #010802, (25)) (abbreviated as GABA del). To inactivate both TH transporters in glutamatergic neurons, Mct8/Oatp1c1 dfl mice were crossed with transgenic animals expressing a constitutively active *Cre*-recombinase under the vesicular glutamate transporter 2 (*Vglut2*) promoter (B6J.129S6 (FVB)-Slc17a6 tm2(cre)Lowl/MwarJ, Jax stock #028863, (26)) (Glut del mice). To monitor Cre recombinase activity, animals additionally harbored a reporter gene insertion consisting of a CAG promoter driven *EYFP* construct within the ROSA26 locus (Gt(ROSA)26Sortm3(CAG-EYFP)Hze) (27). Mct8/Oatp1c1 dfl littermates without *Cre* transgene were used as controls (CTR) to reduce the overall number of generated animals. Animals were analyzed at P12, P21, P60 and P120 to enable comparison with previous studies (18, 22, 28, 29). At P12 and P21, both males and females were collected while at P60 and P120 only male mice were employed in accordance with our published studies on hippocampal function in M/O dKO mice, a model of AHDS (22). If not stated otherwise, animals were sacrificed by cervical dislocation.

To evaluate the deletion of exon 3 in *Mct8* and *Oatp1c1* by PCR studies, brain, liver and kidney samples were collected from P120 animals. Animals were sacrificed and organs were snap-frozen in isopentane on dry ice. For TH serum measurements, animals at P21 were euthanized, blood was collected by heart puncture and serum was isolated through centrifugation. For fluorescent *in situ* hybridization (FISH) studies, mice were sacrificed by cervical dislocation at P12, P21 and P120, and brains were snap-frozen in isopentane on dry ice. For immunofluorescence studies, mice were subjected to intracardial perfusion fixation with 4% phosphate-buffered paraformaldehyde (PFA) in PBS under deep ketamine/xylazine-induced anesthesia at P12, P60 or P120. For EdU-labeling studies, two-month-old mice were injected intraperitoneally with 40 µg/g bw EdU (Thermo Fisher Scientific) and sacrificed 28 days later by intracardial perfusion fixation as above. Whole brains were isolated and post-fixed in 4% PFA for 24 h. For cryo-protection, brains were incubated with 30% sucrose and snap-frozen in isopentane on dry ice. For quantitative real-time PCR (qPCR), animals at P120 were euthanized and hippocampi were macro-dissected and rapidly frozen on dry ice.

Male mice at two months of age were subjected to the pilocarpine model of seizure induction according to published protocols (22). To that end, mice received methyl-scopolamine i.p. (1 mg/kg body weight (bw); Sigma-Aldrich) 30 min prior to the first pilocarpine injection (100 mg pilocarpine hydrochloride/kg bw i.p.; Sigma-Aldrich). Pilocarpine injections were repeated every 20 min until onset of status epilepticus (SE) corresponding to stage 4–5 of the Racine scale characterized by rearing with forelimb clonus (stage 4) and rearing with falling and forelimb clonus (stage 5) (30). After exceeding stage 3 of the Racine scale, mice additionally received a single i.p. injection of xylazine (2.5 mg/kg bw) to reduce their severity burden. 40 min after its detection, SE was terminated by injecting a RISE cocktail i.p. containing Midazolam (1 mg/kg bw; Ratiopharm), MK-801 (0.1 mg/kg bw; Sigma-Aldrich), and 6-methyl-2(phenylethynyl) pyridine hydrochloride (MPEP; 20 mg/kg bw; Sigma-Aldrich). In the recovery phase, mice were kept on a heat pad (30°C), received an i.p. injection of Ringer solution to improve their hydration and were closely monitored. 12 h after SE, mice were subjected to intracardial perfusion-fixation with 4% PFA under deep ketamine/xylazine-induced anesthesia and brains were collected.

### Assessment of TH serum levels

Circulating T4, T3 and rT3 concentrations from CTR, GABA del, Glut del and M/O dKO animals at P21 were quantified in serum samples by LC-MS/MS as described before (31).

### Hybridization-chain-reaction fluorescent *in situ* hybridization

Fresh-frozen coronal forebrain cryo-sections (20 µm) derived from WT animals at P12 and P120 as well as from P21 CTR, GABA del, Glut del and M/O dKO animals were produced between Bregma -1.06 and -3.16 mm with a cryostat (Leica), and pre-treated as described previously (32). For prehybridization, sections were air-dried, fixed for 1 h in a 4% PFA solution (pH 7.4), followed by permeabilization with 0.4% Triton X-100 in PBS for 10 min. Thereafter, sections underwent dehydration by immersion in increasing concentrations of ethanol. Third-generation FISH was performed as previously described (29). Probe sets consisting of 20 individual split-initiator probe pairs targeting *Mct8, Oatp1c1*, Parvalbumin *(Pv)*, Somatostatin *(Sst)*, Calretinin *(Cr)*, Neuropeptide y (*Npy)*, Thyrotropin-releasing hormone (Trh) Krüppel-like factor 9 (Klf9) Neurogranin (Rc3) Crystallin mu (Crym) were employed. Probes and buffers were purchased from Molecular Instruments. Sections were incubated in hybridization buffer for 10 min at 37°C, followed by application of probes (0.4 pmol per 100 µl) in hybridization buffer for 24 h at 37°C. Sections were then washed with probe washing buffer and 5× SSC containing 0.1% Tween-20 (SSCT) according to the manufacturer’s instructions followed by 30 min incubation in amplification buffer at room temperature (RT). Hairpin amplifiers (h1 and h2, 6 pmol per 100 µl amplification buffer) conjugated to Alexa Fluorophores (AxF488, 546, or 647) were heat-denatured at 95°C for 90 s, cooled to RT for 30 min, and then applied onto the slides in amplification buffer at RT. Sections were washed in SSCT, stained with Hoechst 33258 (1:1000; Invitrogen) for 5 min and mounted using Fluoromount (Sigma-Aldrich). Pictures were taken on an AxioObserver.Z1 (Zeiss) microscope or, alternatively, for *Mct8* and *Oatp1c1* expression studies with GABAergic markers, with a Leica SP8 confocal microscope.

### Immunofluorescence studies

Immunofluorescence analyses were performed as previously described (22). Perfusion-fixed brains derived from P12 mice were employed to produce coronal forebrain sections (50 µm) between Bregma -1.06 and -3.64 using a vibratome (Leica). Brain sections were blocked and permeabilized in PBS containing 10% goat serum and 0.2% Triton X-100 (blocking buffer).

Perfusion-fixed, cryo-protected brains from P60 mice subjected to pilocarpine or EdU were cut win a cryostat (Leica, 16 µm thick sections) as described previously (22). Brains from Mct8-KO animals at P12 and P120 employed for βgal staining were processed likewise. Sections were blocked and permeabilized as above. For EdU detection, sections were blocked in 3% BSA in PBS and Click-iT reaction was performed using the Click-iT^®^ EdU Alexa Fluor^®^ 555 Imaging Kit (Thermo Fisher Scientific) according to the manufacturer’s instructions before blocking/permeabilization as above.

Primary antibodies were applied overnight at 4°C in blocking buffer. Subsequently, sections were washed in PBS and incubated for 1 h at RT with Alexa Fluor 488, 555 or 647 labelled secondary antibodies raised in goat (Invitrogen; all 1:1000) and with Hoechst 33258 (Invitrogen; 1:1000) to label cell nuclei in blocking buffer. Sections were washed with PBS and mounted onto microscope slides. Whole brain slice images were scanned using an AxioScan Z.1 (Zeiss) or, alternatively, taken on a Leica SP8 confocal microscope.

The following antibodies were employed: chicken anti-β-galactosidase (1:250, ab9361, Abcam), mouse anti-Gad67 (1:500, MAB5406, Millipore), mouse anti-Parvalbumin (1:500, PV235, Swant), rabbit anti-Somatostatin (1:500, PA5-85759, Invitrogen), rabbit anti-Calretinin (1:500, CR7697, Swant), rabbit anti-Neuropeptide Y (1:1000; #11976, Cell Signalling), mouse anti-Calretinin (1:250, 6B3, Swant), rabbit anti-cFos (1:100; PC05, Calbiochem), chicken anti-GFP (1:500, GFP-1020, Aves), rabbit anti-GABA (1:500, A2052, Sigma-Aldrich), rabbit anti-Prox1 (1:500, AB5475, Sigma-Aldrich), rabbit anti-TBR2/Eomes (1:250, ab23345, Abcam), rabbit anti-Sox2 (1:500, 14-9811-80, Thermo Fisher), chicken anti-Gfap (1:500, 829401, Biolegend), rabbit anti-calbindin (1:500, cb38, Swant), mouse anti-NeuN (1:500, MAB377, Sigma-Aldrich), guinea pig anti-neuronal nuclei (NeuN, 1:1000, 266004, Synaptic Systems), guinea pig anti-doublecortin (1:500, AB2253, Millipore).

### qPCR

For qPCR studies, total RNA was isolated from murine hippocampi using the NucleoSpin RNA Set for NucleoZOL (Macherey-Nagel). cDNA synthesis was performed by reverse transcription using the qScript cDNA SuperMix Kit (Quanta). 10 ng of cDNA were employed for each qPCR replicate. To exclude the presence of genomic DNA, one sample without reverse transcriptase was included as well. qPCR was performed using the qScript™ cDNA SuperMix (Quanta) and a CFX Opus Real-Time PCR Systems (Biorad). Following primers were used: *Gria1*: 5’-CGATAAAGGGGAATGTGGAA-3’ and 5’-TAGCAGAACTCGATTAAGGC-3’; *Gria2*: 5’-ATGGTGGTACGATAAAGGTG-3’ and 5’-TGTAGAATACTCCAGCAACG-3’; *Gria3:* 5’- CCGGCGAATTCTTTTTCTTT-3’ and 5’-AAAAGCCCCAGGACTAAAAA-3’; *Gria4:* 5’-ATAAAGGTGAATGTGGACCC-3’ and 5’-TATGGCTTCGGAAAAAGTCA-3’; *Grik2:* 5’-GAAGGTCTGGAGAAGATTGG-3’ and 5’-CCCATAGAGAGGTTTGTCAG-3’; *Grik4*: 5’-CTACCTTCGCATGGTAGAAT-3’ and 5’-CCATTCCTGGTGAACTGTAA-3’; *Grik5:* 5’-CGAATGGTAGAGTATGACGG-3’ and 5’-TACCACACCCCTATCTCAC-3’; *Grin1*: 5’-TACTCTGACAAGAGCATCCA-3’ and 5’-TTCTCTGCCTTGGACTCA-3’; *Grin2a*: 5’-GACCTCATGACTATTCTCCG-3’ and 5’-AGCTGCGTCATAGATGAAAG-3’; *Grin2b*: 5’-CTGCAAAGAGTCGTAGTGAA-3’ and 5’-GAGGGTTTGAGACGTTTTCT-3’; *Cyclophilin D*: 5’-GCAAGGATGGCAAGGATTGA-3’ and 5’-AGCAATTCTGCCTGGATAGC-3’; *Ppia*: 5’-GTCTTTGGGAAGGTGAAAGA-3’ and 5’-GTCGGAAATGGTGATCTTCT-3’.

### Behavioral studies

For behavioral studies, male mice from CTR, GABA del, Glut del and M/O dKO groups were used at P60. Two weeks prior to the testing, the animals were transferred into a room with reversed light-dark cycle to enable adaptation and perform the tests during the dark/active phase. As tests rely on the natural tendency of rodents to explore the arena and the objects, animals were accustomed to manual handling one week before the testing to reduce stress levels.

The novel object recognition (NOR) test was performed in an open-field arena (50 cm x 50 cm) equipped with a video tracking system (Basler GenICam, acA1300-60). During habituation, each animal was allowed to freely explore the arena for 5 min to reduce possible anxiety-like behaviors. The training session took place 24 h later. Two identical objects (A1 and A2) were placed in opposite corners of the arena and the animal was allowed to explore the objects for 10 min. Interaction with the object was considered when the animal was tilting the head at a distance less than 2 cm from the object. After a retention interval of 24 h, the test was conducted by exchanging one of the familiar objects (A1 or A2) in the arena for a novel object (B1 or B2) (33). The animal was allowed to explore the objects for 10 min and the time spent exploring each object was measured. The total exploration time was calculated by adding the times spent exploring the familiar and novel objects. To avoid object and position bias, both the objects and their positions in the arena were randomized between animals. To avoid olfactory queues, objects and the arena were cleaned with 70% ethanol after each trial. For analysis, the EthoVision XT15 software was used.

For the elevated plus maze (EPM), an elevated maze with a “+”-shaped design consisting of two open arms with a small rim (0.5 cm high) to reduce the probability of falling; and two closed arms with elevated walls (16 cm) was used. The animal was placed on the central platform and allowed to move freely in the maze for 10 min. Movement was recorded by a video camera (Basler GenICam, acA1300-60) above the test apparatus. Subsequently, the time spent in the open/closed arms or the center was determined using the EthoVision XT15 software.

### Quantification

For quantification of cells double positive for βgal and GABAergic markers, the total number of cells positive for Pv, Sst, Cr and Npy were counted followed by enumerating those cells additionally exhibiting βgal immunoreactivity using ZEN software (Zeiss) in the hippocampus (excluding the hilus area and the subgranular zone) and somatosensory cortex. Values were normalized to the size of the area analyzed. Three Hippocampi and somatosensory cortices were analyzed per animal.

For FISH analysis of *Oatp1c1* expression in *Sst*+ interneurons, the total number of *Sst*+ neurons was counted followed by enumerating those cells additionally exhibiting *Oatp1c1* transcripts using ZEN software (Zeiss) in three hippocampus and three somatosensory cortex. Values were normalized to the size of the area analyzed.

Expression of neuron-enriched, TH-regulated target genes (*Klf9*, *Rc3* and *Crym*) was analyzed by FISH in the Cornu ammonis area 1 (CA1) pyramidal layer and in the granule cell layer of the dentate gyrus (DG), where their expression is particularly prominent. Using the ImageJ (NIH) software, mean grey values were recorded and signal-negative areas such as the corpus callosum were used for background subtractions. CTR values were set as 1. Similarly, *Trh* signal intensities were quantified in the paraventricular nucleus (PVN) of the hypothalamus. Three to four pictures of the different brain areas were analyzed per animal.

Immunofluorescence images for the analysis of GABAergic populations were analyzed using the ZEN 3.9 (Zeiss) software. Gad67 staining intensity was quantified by encircling the hippocampus and measuring the mean grey value. Mean grey values in the corpus callosum were measured and subtracted as background. WT values were set as 1.0. Cells positive for Pv, Sst and Cr were counted in whole hippocampi (excluding the hilus area and the SGZ when analyzing Cr+ cells) and normalized to the size of the total analyzed area. Three hippocampal pictures were analyzed per animal.

Evaluation of markers cFos and Sst in the hippocampus CA3 region was performed as before (22). Cells positive for these markers were quantified and normalized to the total area analyzed. Three to four pictures of the CA3 region were analyzed per animal.

To evaluate the hippocampal neurogenic program, images of the DG were acquired using a Leica SP8 confocal microscope. All analyses were performed in the subgranular zone (SGZ) of the DG using ImageJ (NIH) software. Neural stem cells (NSCs) were identified as individual cells double-positive for Sox2 and Gfap and exhibiting a radial process; their numbers were quantified and normalized to the total length of the SGZ within each image. A similar approach was used to quantify transiently amplifying progenitors (TAPs), by counting individual Tbr2+ cells in the SGZ, as well as neuroblasts identified by doublecortin (Dcx) immunoreactivity and immature neurons co-expressing Dcx and Cr. To assess the generation of mature neurons, cells double-positive for EdU and NeuN were quantified in the SGZ, and counts were likewise normalized to the total SGZ length. For NSC, neuroblast and immature neuron evaluation, at least 5 dentate gyrus areas on at least 4 coronal sections of the dorsal hippocampus (Bregma -1.30 to -2.30) were assessed, while for TAPs and the EdU/NeuN co-staining at least 10 areas on 8 sections were investigated.

### Statistics

All data is represented as mean ± SD. Statistical significance between CTR, GABA del, Glut del and M/O dKO mice was evaluated by one-way ANOVA followed by Tukey’s multiple comparison test. Differences were considered significant when p < 0.05 and marked as follows: *, p < 0.05; **, p < 0.01; ***, p < 0.001.

## Results

### Mct8 and Oatp1c1 expression in GABAergic interneurons

**Figure 1:**
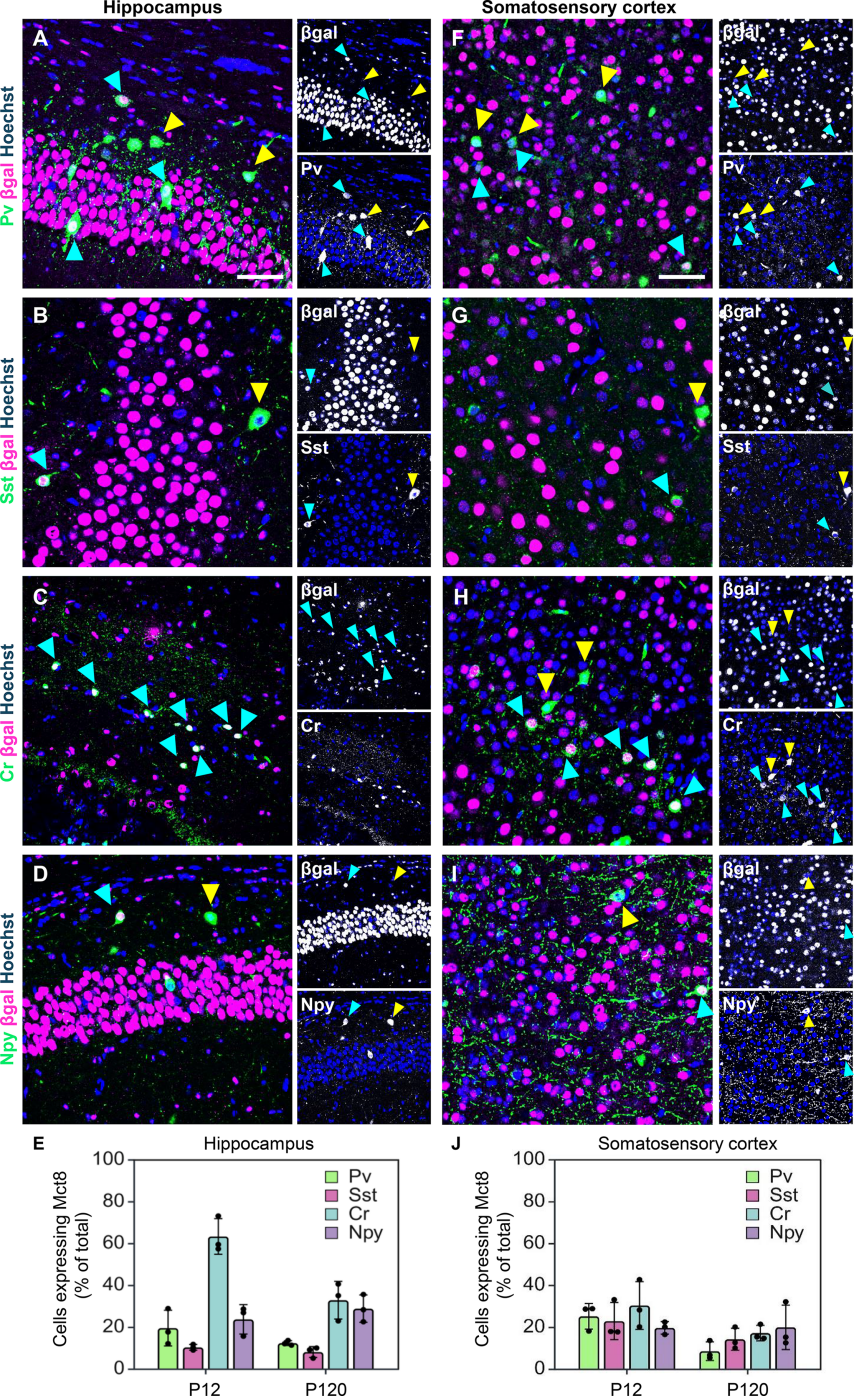
Co-immunofluorescence studies of βgal and GABAergic markers at P12. Perfusion-fixed coronal brain slices from Mct8-KO animals were subjected to immunofluorescence and analyses were performed in the hippocampus **(A-E)** and somatosensory cortex **(F-J)**. Distribution of βgal (magenta) with GABAergic markers (green): Pv **(A, F)**, Sst **(B, G)**, Cr **(C, H)** and Npy **(D, I)** in the hippocampus and somatosensory cortex. Single βgal and GABAergic marker channels are presented in grey scale. Hoechst 33258-stained nuclei are shown in blue in all images. Cyan arrows indicate co-localization of βgal with GABAergic markers and yellow arrows indicate absence of βgal staining in interneurons. The number of cells double positive for βgal and the respective GABAergic marker at P12 and P120 in the hippocampus **(E)** and somatosensory cortex **(J)** was quantified. n = 3. Scale bar: 50 µm.

Mct8 and Oatp1c1 expression have previously been described in murine glutamatergic neurons in both the cerebral cortex and hippocampus (6, 7, 34). To determine whether, and in which GABAergic subtypes these transporters are also expressed, we first assessed Mct8 expression by immunofluorescence using βgal staining in Mct8-KO mice as a proxy of endogenous Mct8 expression (21). βgal staining allows for an unambiguous identification of Mct8-expressing cells as it is observed in nuclei. We then quantified the proportion of βgal-positive cells co-expressing markers of major GABAergic subpopulations, including parvalbumin (Pv), somatostatin (Sst), calretinin (Cr), and neuropeptide Y (Npy) (35). Analyses were performed on perfusion-fixed coronal brain sections from P12 mice (**Figure 1**), focusing on the hippocampal Cornu Ammonis regions CA1 and CA3, as well as the somatosensory cortex (SC).

At P12, βgal staining was prominent in nuclei in both brain areas as expected (**Figure 1**, **Supplementary Figure 1A**). We observed βgal staining in subpopulations of Pv (**Figures 1A, F**), Sst (**Figures 1B, G**), Cr (**Figures 1C, H**) and Npy (**Figures 1D, I**) expressing interneurons in both the hippocampus (**Figures 1A–D**) and SC (**Figures 1F–I**). We then quantified the number of βgal positive cells within the respective interneuron populations both in the hippocampus (**Figure 1E**) and SC (**Figure 1J**). In the P12 hippocampus, Cr+ interneurons represented the population with the highest amount of βgal co-expression (63 ± 8.5%, **Figure 1E**). Moreover, co-staining was present in 19.5 ± 7.1% of Pv+, 10.3 ± 1.9% of Sst+ and 23.7 ± 7.0% of Npy+ interneurons. In contrast, the proportion of GABAergic interneurons exhibiting βgal positivity in the SC was 30.3 ± 11.4% for Cr+, 25.2 ± 6.1% for Pv+, 22.9 ± 8.8% of Sst+ and 19.6 ± 3.0% for Npy+ cells (**Figure 1J**).

We repeated these investigations at P120. In general, a decrease in βgal staining intensity was observed in both the hippocampus and SC (**Supplementary Figure 1B**), with a smaller percentage of βgal+ GABAergic interneurons, in accordance with the well-characterized decrease in Mct8 expression in the adult brain (36). In the hippocampus, βgal immuno-positivity was found in 32.8 ± 9.0% of Cr+, 12.3 ± 1.2% of Pv+, 8.1 ± 2.6% of Sst+ and 28.8 ± 6.5% of Npy+ interneurons (**Figure 1E**). In the SC, we observed βgal staining in 17.1 ± 3.7% of Cr+ interneurons, and in 19.9 ± 10.6% of Npy+ interneurons, whereas only 8.5 ± 4.5% of Pv+ and 14.2 ± 5.2% of Sst+ interneurons were βgal-positive (**Figure 1J**). These findings confirm a heterogeneous expression pattern of Mct8 in GABAergic subpopulation with spatial and temporal differences.

To support these findings, *Mct8* transcripts were assessed by FISH at P12 (**Supplementary Figure 2**). In line with the data above, a subpopulation of *Pv*+ interneurons showed strong *Mct8* mRNA expression in the hippocampus (**Supplementary Figure 2A**). While in the SC the number of *Pv*+ cells with strong *Mct8* expression were seemingly higher, the same heterogeneity existed (**Supplementary Figure 2B**). Likewise, *Sst*+ interneurons showed heterogeneous *Mct8* mRNA expression in the hippocampus (**Supplementary Figure 2C**), whereas in the SC, the number of *Sst+* neurons that exhibited *Mct8* mRNA expression was scarce (**Supplementary Figure 2D**). *Mct8* mRNA was extensively observed in the majority of hippocampal *Cr*+ interneurons (**Supplementary Figure 2E**). However, *Cr*+ interneurons again showed heterogeneous *Mct8* expression in the SC (**Supplementary Figure 2F**). Similarly, in *Npy*+ interneurons, distinct subpopulations showing either strong or absent *Mct8* transcript signals were identified both in the hippocampus (**Supplementary Figure 2G**) and SC (**Supplementary Figure 2H**).

**Figure 2:**
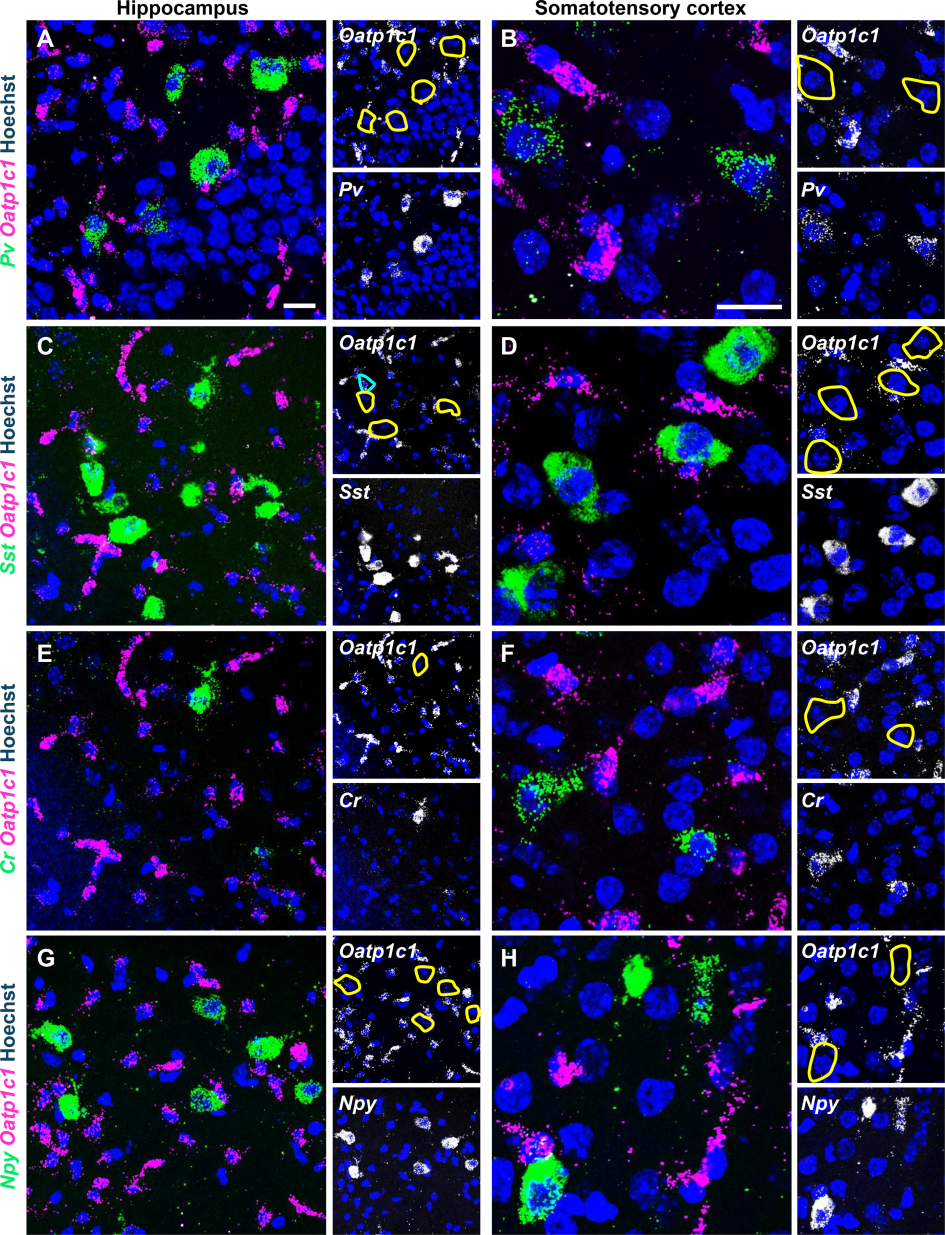
Expression of *Oatp1c1* mRNA in GABAergic interneurons at P12. Fresh-frozen coronal brain slices from wild-type mice were subjected to FISH. Fluorescent images showing *Oatp1c1* mRNA (magenta) co-localization with GABAergic markers (green): *Pv***(A, B)**, *Sst***(C, D)**, *Cr***(E, F)** and *Npy***(G, H)** in the hippocampus **(A, C, E, G)** and somatosensory cortex **(B, D, F, H).** Single *Oatp1c1* and GABAergic marker channels are presented in grey scale. Hoechst 33258-stained nuclei are shown in blue in all images. Cyan circles indicate co-localization of *Oatp1c1* with GABAergic marker while yellow circles indicate absence of *Oatp1c1* expression. n = 4. Scale bar: 20 µm.

We next assessed *Oatp1c1* mRNA expression by FISH using brain slices from P12 animals (**Figure 2**). *Oatp1c1* transcripts were present throughout the brain and strongly localized to vessel-like structures, while low expression was observed in both the pyramidal and granular layers of the hippocampus, as expected (7, 20, 37). No *Oatp1c1* mRNA expression was observed in either *Pv+* (**Figures 2A, B**), *Cr+* (**Figures 2E, F**) or *Npy+* (**Figures 2G, H**) interneurons in the hippocampus and SC. In contrast, *Oatp1c1* mRNA signal was detected in a minor subset of hippocampal *Sst+* cells at P12 (**Figure 2C**, 2.5 ± 0.5% of *Sst+* cells), while in the SC these cells did not exhibit *Oatp1c1* mRNA expression (**Figures 2D, J**). Experiments were repeated at P120 and *Oatp1c1* transcripts were detected in 9.4 ± 3.8% of hippocampal *Sst*+ cells but not in cortical interneurons (pictures not shown).

### Characterization of GABA del and Glut del animals

M/O dKO mice present alterations in hippocampal development and function (22) that are thought to be a consequence of the strongly impaired TH uptake into the brain (18). However, the presence of Mct8 and Oatp1c1 in GABAergic interneurons and glutamatergic neurons suggests an additional, potential role of both TH transporters in providing neurons with TH. To test this hypothesis, two conditional knockout mouse lines were generated harboring a deletion of Mct8/Oatp1c1 either in GABAergic (using Gad2-Cre, GABA del mice) or glutamatergic neurons (using Vglut2-Cre, Glut del mice). As both *Gad2* and *Vglut2* promotor become active around E12.5, the genetic alterations in both mouse models are expected to occur already at early stages of brain development (25, 26, 38–40).

Mice were assessed at different developmental stages in accordance with previous analyses (18, 28, 34). Body weight was recorded, but no differences were observed at either P12 or P21 (**Supplementary Figure 3A**). As control animals for both GABA del and Glut del mice shared the same genotype (Mct8 fl/fl, Oatp1c1 fl/fl, YFP^tg^) and exhibited a similar development, they were combined into a single control group in all subsequent experiments.

Liver, kidney and brain lysates from CTR, GABA del and Glut del mice at P120 were subjected to PCR to confirm a brain-specific exon 3 deletion in the *Slc16a2* and *Slco1c1* genes, as well as the presence of the *Cre* recombinase transgene construct in brain samples of GABA del and Glut del samples (**Supplementary Figure 3**).

Next, Cre expression in the brains of GABA del and Glut del mice was spatially characterized. Perfusion-fixed coronal brain sections of P120 animals were used and Cre expression was traced using YFP reporter staining. In GABA del mice, 93% of YFP+ cells in the SC and hippocampus co-expressed GABA, and 98% of GABA+ cells were YFP+, confirming Cre specificity for GABAergic neurons (**Supplementary Figure 4A**). In contrast, YFP was absent from non-GABAergic neurons such as granular glutamatergic neurons positive for Prox1. YFP was expressed by a small fraction of GABA negative cells (7%, n = 4), consistent with the sporadic YFP expression reported previously in astrocytes (25).

Similarly, in Glut del animals, YFP staining confirmed Cre activity in CA3 pyramidal and DG granule neurons (**Supplementary Figure 4B**). YFP co-localized with Prox1 in the DG and with NeuN throughout the hippocampus, while no signal was observed in GABA-positive or NeuN-negative cells, demonstrating Cre specificity for glutamatergic neurons. Moreover, CTR animals lacking a *Cre* construct showed no YFP staining (**Supplementary Figure 4C**). Therefore, both GABA del and Glut del mice exhibit specific Cre expression in GABAergic and glutamatergic neurons, respectively, excluding major off-target Cre-effects in other cell types.

### Assessment of TH concentrations and TH-dependent genes in GABA del and Glut del mice

**Figure 3:**
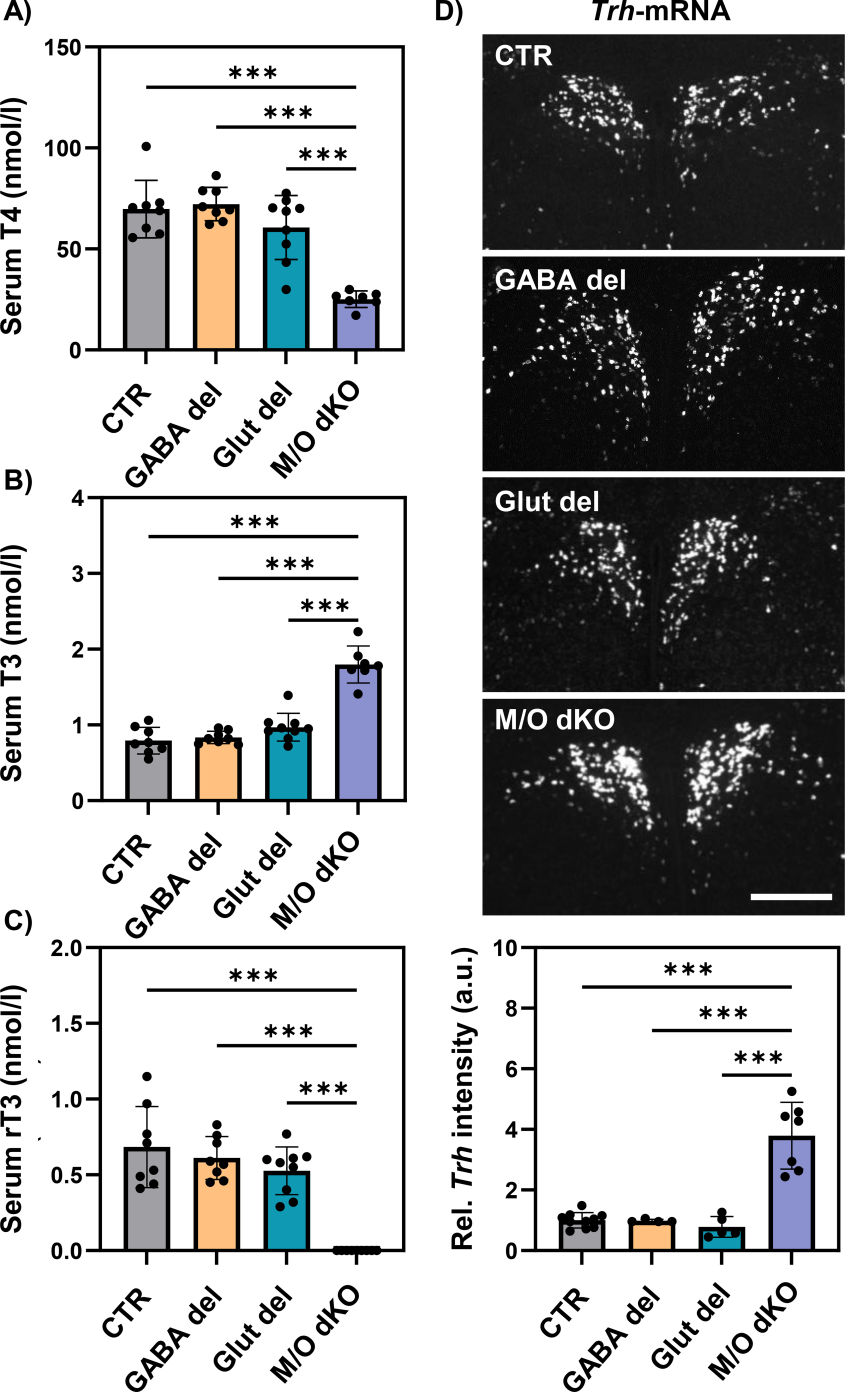
Hypothalamus-pituitary-thyroid axis in GABA del, Glut del and M/O dKO mice at P21. Serum concentrations of total T4 **(A)**, T3 **(B)** and rT3 **(C)** were evaluated by LC-MS/MS. Coronal slices from fresh-frozen brains were subjected to FISH analysis and *Trh* transcript levels were evaluated in the PVN by determining the mean grey values **(D)** in the different experimental groups: control (CTR), GABA del, Glut del and Mct8/Oatp1c1 dKO (M/O dKO) mice. CTR values were set as 1. n = 6–8 mice per genotype. ***p<0.001. Scale bar: 300 µm.

Global absence of Mct8 and Oatp1c1 is known to result in abnormal serum TH profile characterized by elevated T3, decreased T4 and rT3 concentrations below detection limits (18, 41). At P21, serum concentrations of T4 (**Figure 3A**), T3 (**Figure 3B**) and rT3 (**Figure 3C**) in GABA del and Glut del were comparable to those of CTR animals, while M/O dKO mice displayed the expected alterations.

**Figure 4:**
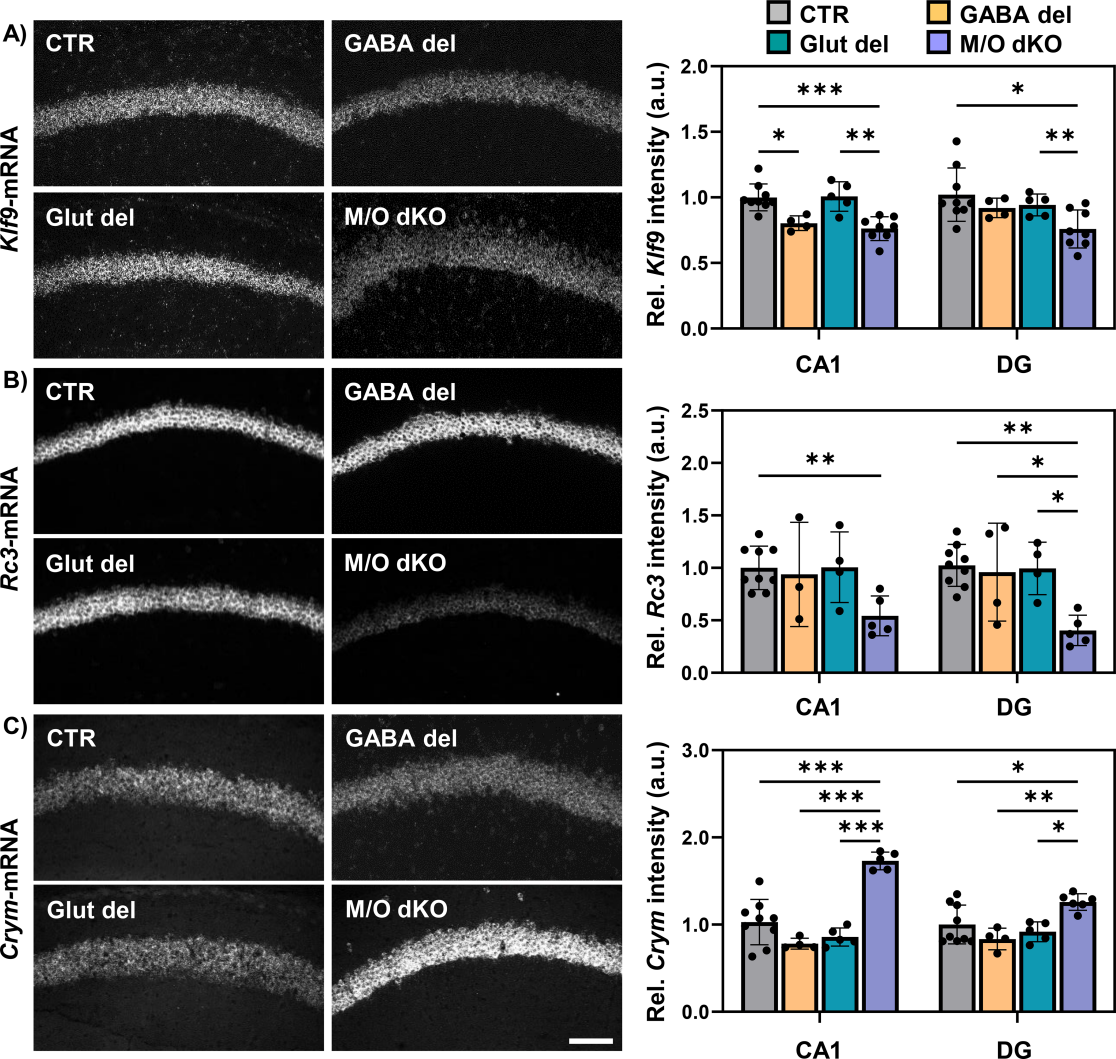
Evaluation of TH regulated genes in GABA del, Glut del and M/O dKO mice at P21. Fresh-frozen coronal brain sections were used for FISH analysis of TH-target gene expression. Representative images of the hippocampal CA1 region and mean grey values of *Klf9***(A)**, *Rc3***(B)** and *Crym***(C)** in the CA1 and DG in the different experimental groups: control (CTR), GABA del, Glut del mice and Mct8/Oatp1c1 dKO (M/O dKO) mice. CTR values were set as 1. n = 3–9 mice per genotype. *p<0.05, **p<0.01, ***p<0.001. Scale bar: 100 µm.

The expression of well-known TH-regulated genes was evaluated in different brain regions at P21. Fresh-frozen coronal brain sections were used to analyze levels of *Trh* mRNA in the PVN of the hypothalamus by FISH (**Figure 3D**). The intensity of the *Trh* signal was measured, but no differences were found in either GABA del or Glut del mice compared to CTR animals, while *Trh* transcripts were increased in M/O dKO mice. Additionally, neuron-enriched TH-regulated genes were evaluated specifically in hippocampal CA1 and DG areas. *Klf9* expression, which is positively regulated by TH, was comparable between Glut del and CTR animals, while *Klf9* levels in the CA1 region were decreased in GABA del animals (**Figure 4A**). However, normal expression levels of *Klf9* were observed in the DG, suggesting region-specific differences. In contrast, the positively TH-regulated gene *Rc3* (**Figure 4B**) and the negatively TH-regulated gene *Crym* (**Figure 4C**) did not show differences in transcript levels between GABA del, Glut del and CTR mice in the CA1 and DG areas. M/O dKO mice exhibited the expected alterations in all FISH experiments. These findings indicate that Mct8 and Oatp1c1 deletion in either the GABAergic or in the glutamatergic system does not alter TH homeostasis or TH action in neurons.

### Evaluation of GABAergic populations and expression of ionotropic glutamatergic receptor subunits in GABA del and Glut del animals

**Figure 5:**
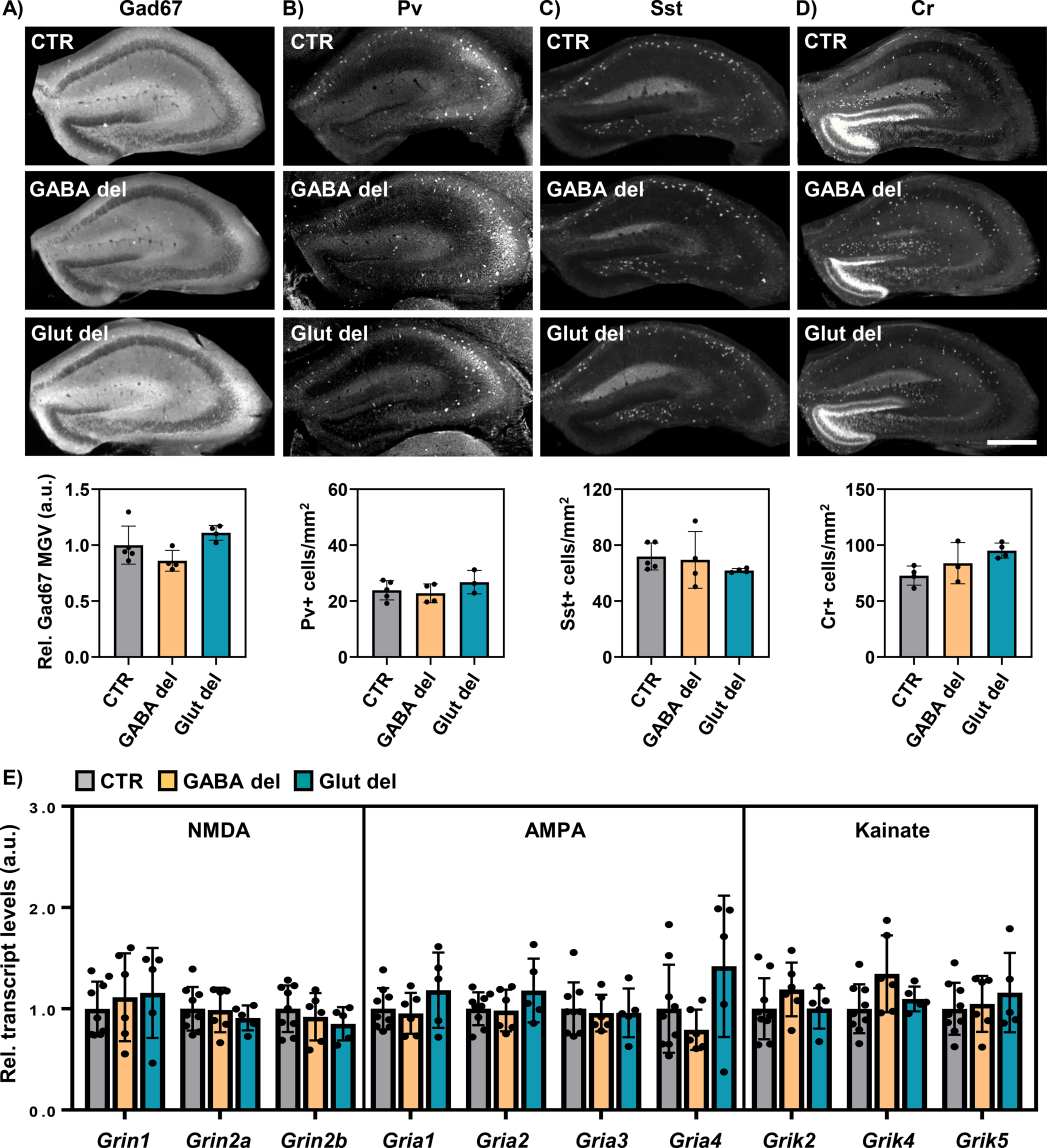
Evaluation of GABAergic subpopulations and ionotropic glutamatergic receptor subunits in GABA del and Glut del mice. Coronal forebrain vibratome sections of P12 mice were subjected to immunofluorescence analyses. Representative images of hippocampal sections stained with Gad67 and evaluation of Gad67 intensity by mean grey values (MGV) measurement in the hippocampus **(A)**. CTR values set as 1. Representative images and quantification of cells positive for Pv **(B)**, Sst **(C)** and Cr **(D)** at P12. Hippocampal homogenates were subjected to qPCR analysis and expression of NMDA, AMPA and kainate **(E)** receptor subunits was assessed at P120 in the different experimental groups: control (CTR), GABA del and Glut del mice. Expression levels were normalized to *CycloD* and *Ppia* expression as housekeeping genes and CTR values were set as 1. n = 4–9 animals per genotype.

Previous studies from our lab revealed alterations in GABAergic hippocampal interneuron subpopulations in global Mct8/Oatp1c1 deficiency, with major differences observed at P12 (22). To determine whether the absence of both TH transporters in GABAergic or glutamatergic neurons similarly affects GABAergic neurodevelopment, the same markers that showed alterations in M/O dKO mice before were investigated. Protein expression of the general GABAergic marker glutamic acid decarboxylase 67 (Gad67), a GABA-synthesizing enzyme, was evaluated but no differences in staining intensity were observed between GABA del, Glut del and CTR animals (**Figure 5A**). GABAergic subpopulation numbers positive for Pv (**Figure 5B**), Sst (**Figure 5C**) and Cr (**Figure 5D**) were also similar in all experimental groups.

Another parameter strongly affected in global M/O dKO mice is the expression of multiple subunits of ionotropic glutamate receptors in the hippocampus at P120 (22). Here, qPCR analyses were performed employing total hippocampal mRNA isolates from CTR, GABA del and Glut del mice at P120 (**Figure 5E**). No significant differences were found among animal groups for any of the NMDA, AMPA or kainate receptor subunits analyzed. Overall, these results indicate an unaltered GABAergic and glutamatergic systems development after neuron-specific deletion of Mct8 and Oatp1c1.

### Evaluation of seizure susceptibility in GABA del and Glut del animals

**Figure 6:**
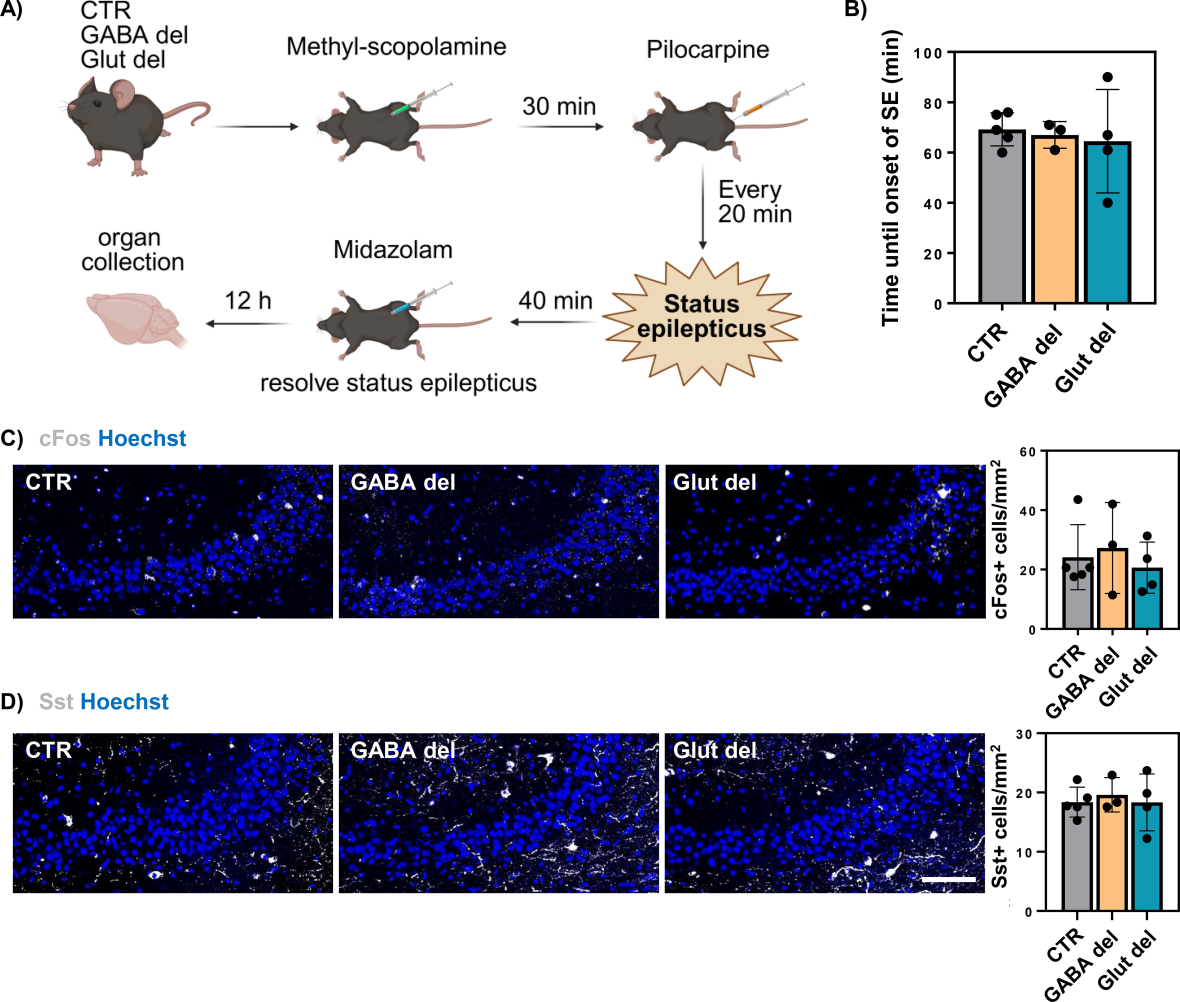
Seizure susceptibility in GABA del and Glut del mice. Schematic representation of the pilocarpine model of seizure induction **(A)**. Time until onset of SE after pilocarpine injection in CTR, GABA del and Glut del mice at P60 **(B)**. Immunohistochemical evaluation of brains subjected to pilocarpine 12 h after SE. Representative images and quantification of cells positive for markers cFos **(C)** and Sst **(D)** (in white) in the hippocampal CA3 of the different experimental groups subjected to pilocarpine: control (CTR), GABA del and Glut del mice. Hoechst 33258-stained nuclei are shown in blue in all images. n = 3–5 animals per genotype. Scale bar: 100 µm.

To assess putative functional consequences, GABA del and Glut del animals were subjected to the convulsant agent pilocarpine (**Figure 6A**), as previously described (22). No differences were observed in the time required for SE onset nor did GABA del or Glut del mice exhibit an increased mortality (**Figure 6B**), while M/O dKO mice are known to reach SE faster and possess a higher mortality rate after seizure induction (22). Additionally, no differences in the expression of the neuronal activation marker cFos were found in GABA del or Glut del mice in comparison to CTR animals subjected to pilocarpine 12 h after SE (**Figure 6C**), unlike seen before in M/O dKO mice. Similarly, Sst expression remained unaffected in GABA del or Glut del mice (**Figure 6D**). Altogether, these findings indicate that Mct8 and Oatp1c1 deletion in GABAergic and glutamatergic neurons does not influence neuronal excitability or seizure susceptibility.

### Assessment of the adult neurogenic program in the subgranular zone in GABA del and Glut del animals

Previous evaluations from our group revealed a key role of Mct8 and Oatp1c1 in the process of hippocampal neurogenesis which takes place in the SGZ of the DG and is essential for proper hippocampal function (34, 42). In this process, neural stem cells (NSCs) positive for the markers Sox2, Gfap and harboring a radial process extending into the granule cell layer proliferate and give rise to transiently amplifying progenitors (TAPs) expressing the marker Tbr2. TAPs progress into neuroblasts that upregulate Doublecortin (Dcx), exit the cell cycle and become immature neurons, which in addition to Dcx express (Cr). These immature cells then mature over 4–6 weeks to new granule cell neurons (43). As GABAergic and glutamatergic signaling influence the progression through the adult hippocampal neurogenic program (44), we evaluated whether this process was altered when Mct8 and Oatp1c1 were deleted in both neuronal populations.

**Figure 7:**
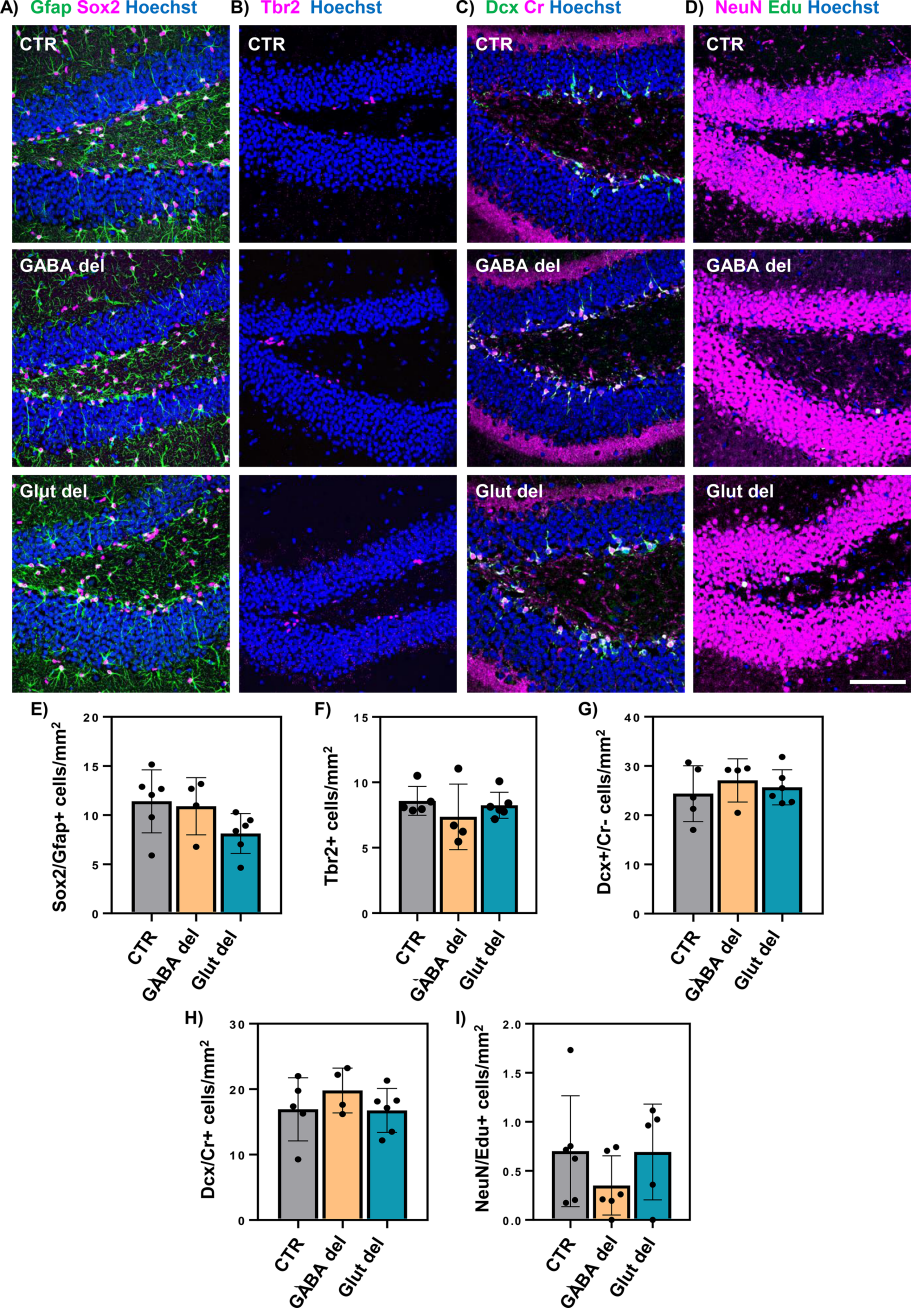
Assessment of the neurogenic program in the hippocampal subventricular zone in GABA del and Glut del animals. Perfusion-fixed coronal brain slices were subjected to immunofluorescence analyses. Representative pictures of cells positive for Sox2 (magenta) and Gfap (green) **(A)**, Trb2 (magenta) **(B)**, doublecortin (Dcx, green) and calretinin (Cr, magenta) **(C)**, and NeuN (magenta) and EdU (green) **(D)** in the DG of the hippocampus. Quantification of the number of cells double positive for Sox2 and Gfap **(E)**, for Tbr2 **(F)**, for Dcx and negative for Cr **(G)**, double positive for Dcx and Cr **(H)** and double positive for NeuN and EdU **(I)** per mm in the subgranular zone of the DG. Hoechst 33258-stained nuclei are shown in blue in all images. Data presented as number of positive cells per mm. n = 4-6. Scale bar: 100 µm.

For this purpose, we characterized the different stages of progenitor proliferation and maturation in the SGZ of P60 GABA del and Glut del mice by immunofluorescence using perfusion-fixed coronal brain slices (**Figure 7**). The number of NSCs were unaltered in all experimental groups (**Figures 7A, E**). Similarly, no differences were observed in TAPs (**Figures 7B, F**), in neuroblasts (**Figures 7C, G**) and in immature neurons (**Figures 7C, H**) in GABA del or Glut del mice. Finally, 28 days after EdU injection, a similar number of mature neurons (positive for EdU and NeuN) was observed across all experimental groups, though minor alterations may be masked by distinct intra-group variability (**Figures 7D, I**). Together, these data do not support a pronounced effect of Mct8/Oatp1c1 deletion in GABAergic or glutamatergic neurons on adult hippocampal neurogenesis.

### Hippocampus-dependent behavior analyses

**Figure 8:**
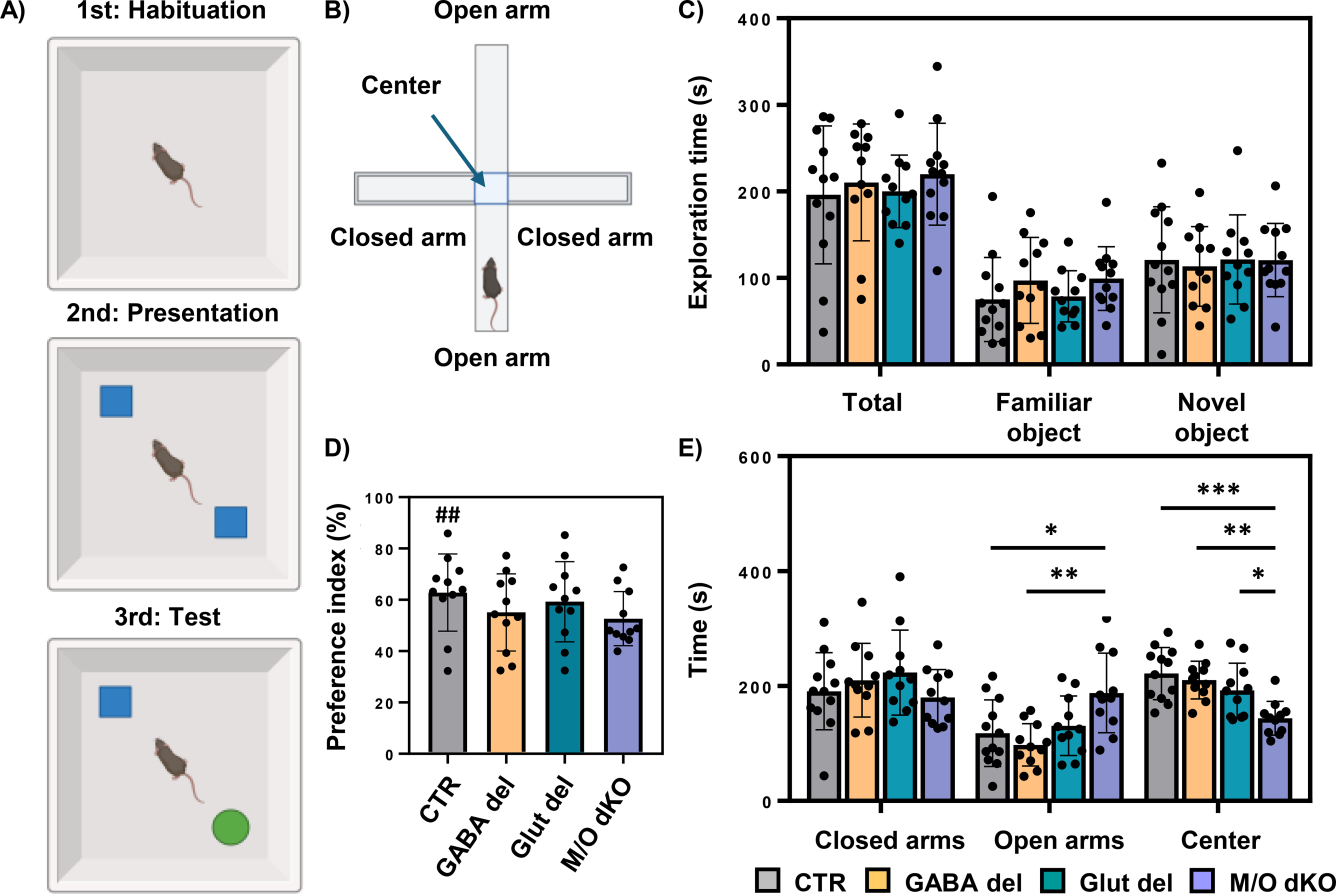
Hippocampus-dependent behavior. Schematic representation of the novel object recognition (NOR) protocol: first day (habituation), second day (presentation of the objects) and third day (test) **(A)**. Schematic representation of the elevated plus maze (EPM) set-up **(B)**. The total time exploring the objects in seconds (s), and the time spent exploring the familiar and novel object was evaluated **(C)**. Preference index, calculated as the time spent exploring the novel object divided by the total exploration time and multiplied by 100. The groups significantly exceeding chance levels (50%) are indicated. ##, p < 0.01 **(D)**. Time in seconds (s) spent by the animals in the closed and open arms and in the center of the maze **(E)** in the different experimental groups: control (CTR), GABA del, Glut del and Mct8/Oatp1c1 dKO (M/O dKO). n = 10-12 mice per genotype. *p<0.05, **p<0.01, ***p<0.001.

To further evaluate hippocampus-related functional aspects, GABA del and Glut del mice at P60 were subjected to the NOR (**Figure 8A**) and EPM tests (**Figure 8B**) to analyze object recognition and anxiety-like behaviors, respectively. As these tests were not performed before with M/O dKO mice, these animals were included as well for comparison.

In the NOR test, no differences were observed in the total exploration time for both objects between groups (**Figure 8C**). Similarly, no differences were found in the time spent exploring the novel and familiar object across all animal groups. Notably, the preference index (**Figure 8D**), calculated as the time spent exploring the novel object divided by the total exploration time × 100, was significantly above chance level (50%) only in CTR animals, indicating that the other groups did not show a clear preference for the novel object. However, no significant differences in preference index were detected between genotypes. Neither cell type–specific nor global deletion of Mct8/Oatp1c1 appeared to affect NOR performance compared with CTR animals.

Next, anxiety-like behaviors were assessed in the EPM test with the same GABA del, Glut del and M/O dKO mice used above. No differences were observed among the different animal groups in the time spent in the closed arms (**Figure 8E**). However, M/O dKO mice notably spent more time in the open arms but less time in the center of the maze, while no such differences were seen in GABA del or Glut del mice. These findings suggest that the conditional deletion of Mct8 and Oatp1c1 in the GABAergic and glutamatergic systems does not impact anxiety, while the global deletion of these transporters alters anxiety-like behaviors in M/O dKO mice during the EPM.

## Discussion

A prerequisite for TH action in both the developing and mature CNS is the presence of TH transporters at brain barriers that ensure TH availability within the brain and in target cells. Thus, it is not surprising that MCT8 deficiency in humans, which is thought to impair TH uptake across brain barriers and cause a severe central TH deficiency, results in an abnormal neurodevelopment that leads to microcephalia, absence of speech development, low IQ and epileptic seizures (15–17). Similarly, alterations in neuronal development were also reported in the cerebral cortex and hippocampus of M/O dKO mice, an established murine model for human MCT8 deficiency (18, 22). The importance of Mct8 and Oatp1c1 at the blood–brain barrier (BBB) was recently underscored by a study showing that a mouse model with conditional deletion of both transporters in BBB endothelial cells recapitulates the neuronal alterations observed in global M/O double-knockout mice (41). Notably, these abnormalities were less severe than those observed in M/O dKO mice, suggesting that additional TH transport across other brain barriers and/or neuronal TH uptake in this model partially mitigate the severe phenotype of global M/O dKO mice.

While the expression of both Mct8 and Oatp1c1 was shown before in pyramidal neurons in the murine cerebral cortex and hippocampus (6, 7, 10, 34, 36), information about their distribution in GABAergic interneurons in mice is scarce. Here, we provide a comprehensive analysis of Mct8 and Oatp1c1 expression in major GABAergic interneuron subpopulations in both the hippocampus and SC. We revealed a similarly heterogenous distribution of Mct8 in Pv+ interneurons in the hippocampus and cerebral cortex. These differences may reflect the intrinsic heterogeneity within the Pv+ subpopulation, which can itself be subdivided into chandelier, basket, and translaminar cell types (45, 46). Likewise, heterogeneity in Mct8 expression in subsets of Sst+, Cr+, and Npy+ interneurons was observed, as well as in *Oatp1c1* mRNA expression, which was only observed in some *Sst*+ interneurons. These patterns further highlight the substantial molecular diversity within major GABAergic interneuron classes. To pinpoint which specific Pv and Sst interneuron subtypes express Mct8 and Oatp1c1, future work integrating morphological and electrophysiological characterization will be required.

Recently, protein expression of both MCT8 and OATP1C1 was addressed in the adult macaque and human motor cortex (8, 11). MCT8 was reported in SST+ and CR+ interneurons in humans and in PV+ and SST+ interneurons in macaque, consistent with our findings in the murine brain. OATP1C1, in contrast, was widely expressed across GAD+ interneurons and specifically co-localized to CR+ and Calbindin+ interneurons in the macaque cerebral cortex, which contrasts with the murine data presented here and points to species-specific differences. Additionally, the authors reported MCT8 and OATP1C1 expression in pyramidal neurons in the adult human and macaque cortex (8). Therefore, a potential role of both MCT8 and OATP1C1 in regulating TH availability in neurons not only in mice, but also in macaques and humans can be hypothesized.

To address a putative neuron-specific impact of Mct8 and Oatp1c1, two mouse models with deletion of both TH transporters either in all GABAergic or glutamatergic neurons were generated.

Serum TH concentrations as well as the expression of TH-dependent genes in the hippocampus and PVN remained unchanged in both GABA del and Glut del mice, indicating an intact hypothalamus-pituitary-thyroid axis and a normal TH sensing in neurons. Putative compensatory changes preserving TH signaling in neurons remain elusive. Similarly, neither the development of the inhibitory GABAergic system in the hippocampus nor the expression of ionotropic glutamate receptor subunits therein differed from control animals. This contrasts previously published data on M/O dKO mice hippocampi, which showed reduced Gad67 levels and Pv+ interneuron numbers as well as increased Sst+ neurons at P12, and higher expression of glutamate receptor subunits in adulthood (22). Moreover, M/O dKO mice exhibited signs of heightened network excitability, evidenced by a markedly shorter latency to pilocarpine-induced SE (~40 min after first injection), while GABA del and Glut del mice required approximately 70 min to reach SE, comparable to controls. Thus, neuron-specific absence of Mct8 and Oatp1c1 does not seem to perturb the timed development or function of the GABAergic and glutamatergic systems.

The unaltered GABAergic interneuron development in GABA del mice differs from our previous observations made in a mouse model lacking Mct8 and Oatp1c1 in Nkx2.1- expressing cells (Nkx2.1 del mice) (29). In the developing brain, Nkx2.1 is active in the medial ganglionic eminence, where it is critical for Pv+ and Sst+ interneuron generation (47). At P12, Nkx2.1 del mice exhibited transiently reduced Gad67 expression and changes in Pv+ and Cr+ interneuron subpopulations in the somatosensory cortex, suggesting a cell-autonomous function of Mct8/Oatp1c1 in these subsets of GABAergic interneurons (29). The discrepancy between GABA del and Nkx2.1 del animals may be due to spatial differences as here we focused on the hippocampus. It is therefore possible that GABAergic subpopulations display a region-specific, cell-autonomous requirement for Mct8 and Oatp1c1 during development and in adulthood in agreement with our findings in global M/O dKO mice (18, 22).

Along this line, the cell-autonomous importance of TH for GABAergic interneuron development was demonstrated before in a mouse model harboring a dominant negative TRα1 mutation, which inhibits the receptor’s transcriptional activity, in all GABAergic neurons (48). The authors employed the same Gad2-Cre construct as we used in this study, and reported a reduction in Pv+ interneurons accompanied by an increase in Sst+ interneurons in the hippocampus at P14, as well as spontaneous seizures and early mortality within the first three postnatal weeks, emphasizing the critical role of TH receptor signaling in GABAergic interneurons.

The hippocampus plays a central role in both memory and emotional behaviors, functions closely tied to the generation of new neurons through adult hippocampal neurogenesis (49, 50). Using the well-established NOR and EPM paradigms to assess memory recall and anxiety, respectively, no behavioral abnormalities were observed in GABA del or Glut del mice at P60. A potential limitation is that testing was performed after a 24-hour retention interval, and the preference index in GABA del, Glut del or M/O dKO mice did not exceed chance level, indicating possible memory decay. Subtle genotype-dependent differences may thus be detectable only at shorter retention intervals. The absence of behavioral alterations in GABA del and Glut del mice is further supported by the unaltered adult hippocampal neurogenic program observed in these mice, consistent with preserved hippocampal function. Contrary, other models with disrupted TH signaling in all neurons or specially in GABAergic neurons, showed an increased anxiety-like behavior (48, 51). These findings further support the notion that while TH action in neurons is essential, Mct8 and Oatp1c1 expression therein is dispensable for hippocampus-dependent functions.

Similarly, M/O dKO mice showed no behavioral differences compared to CTR animals during the NOR paradigm. However, M/O dKO animals displayed decreased anxiety-like behavior in the EPM, where mice preferentially occupied the open arms. This contrasts with previous results in which an increased anxiety in M/O dKO mice was observed in the open-field test (34, 52). This discrepancy may reflect differences in the test paradigm, as well as the age of the animals or activity phase in which the mice were tested. Yet, the reduction in EPM-associated anxiety-like behavior reported here aligns with observations in congenitally hypothyroid rat models (53, 54). While the EPM evaluates primarily anxiety, alterations in exploratory behaviors, spatial learning, avoidance, and overall mobility can also influence its outcome (55). Notably, M/O dKO mice exhibit deficits in spatial learning and memory at P30 in the Barnes maze, as well as reduced spontaneous alternations in the Y-maze (56). These alterations in exploratory behavior and spatial cognition in M/O dKO mice may therefore contribute to the increased time spent in the open arms of the EPM observed here.

Together, the data presented here allow us to conclude that Mct8 and Oatp1c1 do not play a major cell-autonomous role in the development and function of the GABAergic and glutamatergic neurons in the hippocampus. Instead, THs likely enter neurons in GABA del and Glut del mice via other TH transporters such as Mct10, Lat1 or Lat2 in the absence of Mct8/Oatp1c1 (10, 57, 58). Our findings substantiate the critical role of Mct8/Oatp1c1 in TH uptake at brain barriers and in ensuring adequate TH availability for neuronal development and function (18, 41, 59). Although these observations require validation in the human system, they are informative for developing therapeutic strategies for MCT8 deficiency. In this respect, restoring MCT8 expression at brain barriers through gene therapy (56, 60) appears as a promising strategy to increase TH availability in the brain and thereby improve neurological parameters in MCT8 deficient patients.

## Data Availability

The raw data supporting the conclusions of this article will be made available by the authors, without undue reservation.

## Supplementary material

Supplementary Figure 1 βgal expression in Mct8-KO animals. Perfusion-fixed coronal brain slices from Mct8-KO animals were subjected to immunofluorescence analysis. Representative pictures of βgal staining (magenta) and Hoechst 33258-stained nuclei (blue) in the somatosensory cortex (SC) and CA3 area of the hippocampus of mice at P12 **(A)** and P120 **(B)**. Scale bar: 100 µm.

Supplementary Figure 2 Co-expression of *Mct8* mRNA with GABAergic markers in wild-type hippocampus at P12. Fresh frozen coronal brain slices were subjected to FISH. Fluorescent images showing *Mct8* mRNA (in magenta) co-localization with *Pv***(A, B)**, *Sst***(C, D)**, *Cr***(E, F)** and *Npy***(G, H)** (all in green) in the hippocampus **(A, C, E, G)** and somatosensory cortex **(B, D, F, H)**. Single *Mct8* and GABAergic marker channels are presented in grey scale. Hoechst 33258-stained nuclei are shown in blue in all images. Cyan circles indicate co-localization of *Mct8* with GABAergic marker while yellow circles indicate absence of *Mct8* expression. n = 4. Scale bar: 20 µm.

Supplementary Figure 3 Evaluation of conditional animals. The body weight of the animals was recorded at postnatal days P12 and P21 **(A)**. Genomic DNA derived from liver, kidney and brain homogenates of control (CTR), GABA del and Glut del mice at P120 was subjected to PCR analysis. Detection of Mct8 (M8) **(B)** and Oatp1c1 (O1) **(C)** flox (fl) and del alleles in different tissues. Confirmation of Cre **(D)** and YFP **(E)** transgene presence in brains of the different experimental animals. M, molecular-sized weight marker in base pairs (bp); WT, wild-type allele.

Supplementary Figure 4 YFP immunostaining in hippocampal sections of GABA del and Glut del mice at P120. Representative immunohistochemical images of coronal vibratome sections depicting YFP (green) as an indicator of Cre recombination. Co-staining with antibodies against GABA, Prox1, or NeuN (all shown in magenta) was performed to visualize GABAergic, glutamatergic, or general neuronal populations, respectively, in the hippocampal CA3 or DG regions in GABA del **(A)** and Glut del mice **(B)**. Representative immunofluorescence images of YFP in control (CTR) mice with no Cre activity **(C)**. Hoechst33258-stained nuclei are shown in blue in all images. Scale bar: 100 µm.

## References

[1] Alcaide Martin A Mayerl S . Local thyroid hormone action in brain development. Int J Mol Sci. (2023) 24. doi: 10.3390/ijms241512352, PMID: 37569727 PMC10418487

[2] Bernal J . Thyroid hormones and brain development. Vitam Horm. (2005) 71:95–122. doi: 10.1016/S0083-6729(05)71004-9, PMID: 16112266

[3] Flamant F Gauthier K . Thyroid hormone receptors: the challenge of elucidating isotype-specific functions and cell-specific response. Biochim Biophys Acta. (2013) 1830:3900–7. doi: 10.1016/j.bbagen.2012.06.003, PMID: 22704954

[4] Bernal J . Thyroid and Brain understanding the actions of thyroid hormones in brain development and Function. Bentham Science Publishers: Sharjah (2024).

[5] Groeneweg S Geest V Peeters RP Heuer H Visser WE . Thyroid hormone transporters. Endocr Rev. (2020) 41:146–201. doi: 10.1210/endrev/bnz008, PMID: 31754699

[6] Heuer H Maier MK Iden S Mittag J Friesema EC Visser TJ . The monocarboxylate transporter 8 linked to human psychomotor retardation is highly expressed in thyroid hormone-sensitive neuron populations. Endocrinology. (2005) 146:1701–6. doi: 10.1210/en.2004-1179, PMID: 15661862

[7] Muller J Heuer H . Expression pattern of thyroid hormone transporters in the postnatal mouse brain. Front Endocrinol (Lausanne). (2014) 5:92. doi: 10.3389/fendo.2014.00092, PMID: 24994998 PMC4061481

[8] Wang Y Wang T Montero-Pedrazuela A Guadano-Ferraz A Rausell E . Thyroid hormone transporters MCT8 and OATP1C1 are expressed in pyramidal neurons and interneurons in the adult motor cortex of human and macaque brain. Int J Mol Sci. (2023) 24. doi: 10.3390/ijms24043207, PMID: 36834621 PMC9965431

[9] Lopez-Espindola D Garcia-Aldea A Gomez De La Riva I Rodriguez-Garcia AM Salvatore D Visser TJ . Thyroid hormone availability in the human fetal brain: novel entry pathways and role of radial glia. Brain Struct Funct. (2019) 224:2103–19. doi: 10.1007/s00429-019-01896-8, PMID: 31165302

[10] Wirth EK Roth S Blechschmidt C Holter SM Becker L Racz I . Neuronal 3’,3,5-triiodothyronine (T3) uptake and behavioral phenotype of mice deficient in Mct8, the neuronal T3 transporter mutated in Allan-Herndon-Dudley syndrome. J Neurosci. (2009) 29:9439–49. doi: 10.1523/JNEUROSCI.6055-08.2009, PMID: 19641107 PMC6666526

[11] Wang T Wang Y Montero-Pedrazuela A Prensa L Guadano-Ferraz A Rausell E . Thyroid hormone transporters MCT8 and OATP1C1 are expressed in projection neurons and interneurons of basal ganglia and motor thalamus in the adult human and macaque brains. Int J Mol Sci. (2023) 24. doi: 10.3390/ijms24119643, PMID: 37298594 PMC10254002

[12] Dumitrescu AM Liao XH Best TB Brockmann K Refetoff S . A novel syndrome combining thyroid and neurological abnormalities is associated with mutations in a monocarboxylate transporter gene. Am J Hum Genet. (2004) 74:168–75. doi: 10.1086/380999, PMID: 14661163 PMC1181904

[13] Friesema ECH Grueters A Biebermann H Krude H Von Moers A Reeser M . Association between mutations in a thyroid hormone transporter and severe X-linked psychomotor retardation. Lancet. (2004) 364:1435–7. doi: 10.1016/S0140-6736(04)17226-7, PMID: 15488219

[14] Schwartz CE May MM Carpenter NJ Rogers RC Martin J Bialer MG . Allan-Herndon-Dudley syndrome and the monocarboxylate transporter 8 (MCT8) gene. Am J Hum Genet. (2005) 77:41–53. doi: 10.1086/431313, PMID: 15889350 PMC1226193

[15] Groeneweg S Van Geest FS Abaci A Alcantud A Ambegaonkar GP Armour CM . Disease characteristics of MCT8 deficiency: an international, retrospective, multicentre cohort study. Lancet Diabetes Endocrinol. (2020) 8:594–605. doi: 10.1016/S2213-8587(20)30153-4, PMID: 32559475 PMC7611932

[16] Lopez-Espindola D Morales-Bastos C Grijota-Martinez C Liao XH Lev D Sugo E . Mutations of the thyroid hormone transporter MCT8 cause prenatal brain damage and persistent hypomyelination. J Clin Endocrinol Metab. (2014) 99:E2799–804. doi: 10.1210/jc.2014-2162, PMID: 25222753 PMC4255116

[17] Remerand G Boespflug-Tanguy O Tonduti D Touraine E Rodriguez D Curie A . Expanding the phenotypic spectrum of Allan-Herndon-Dudley syndrome in patients with SLC16A2 mutations. Dev Med Child Neurol. (2019) 61:1439–47. doi: 10.1111/dmcn.14332, PMID: 31410843

[18] Mayerl S Muller J Bauer R Richert S Kassmann CM Darras VM . Transporters MCT8 and OATP1C1 maintain murine brain thyroid hormone homeostasis. J Clin Invest. (2014) 124:1987–99. doi: 10.1172/JCI70324, PMID: 24691440 PMC4001533

[19] Dumitrescu AM Liao XH Weiss RE Millen K Refetoff S . Tissue-specific thyroid hormone deprivation and excess in monocarboxylate transporter (mct) 8-deficient mice. Endocrinology. (2006) 147:4036–43. doi: 10.1210/en.2006-0390, PMID: 16709608

[20] Roberts LM Woodford K Zhou M Black DS Haggerty JE Tate EH . Expression of the thyroid hormone transporters monocarboxylate transporter-8 (SLC16A2) and organic ion transporter-14 (SLCO1C1) at the blood-brain barrier. Endocrinology. (2008) 149:6251–61. doi: 10.1210/en.2008-0378, PMID: 18687783

[21] Trajkovic M Visser TJ Mittag J Horn S Lukas J Darras VM . Abnormal thyroid hormone metabolism in mice lacking the monocarboxylate transporter 8. J Clin Invest. (2007) 117:627–35. doi: 10.1172/JCI28253, PMID: 17318265 PMC1797602

[22] Alcaide Martin A Bauer R Fuhrer-Sakel D Heuer H Mayerl S . Increased seizure susceptibility in thyroid hormone transporter Mct8/Oatp1c1 knockout mice is associated with altered neurotransmitter systems development. Prog Neurobiol. (2025) 247:102731. doi: 10.1016/j.pneurobio.2025.102731, PMID: 39986448

[23] Mayerl S Schmidt M Doycheva D Darras VM Huttner SS Boelen A . Thyroid hormone transporters MCT8 and OATP1C1 control skeletal muscle regeneration. Stem Cell Rep. (2018) 10:1959–74. doi: 10.1016/j.stemcr.2018.03.021, PMID: 29706500 PMC5993536

[24] Mayerl S Visser TJ Darras VM Horn S Heuer H . Impact of oatp1c1 deficiency on thyroid hormone metabolism and action in the mouse brain. Endocrinology. (2012) 153:1528–37. doi: 10.1210/en.2011-1633, PMID: 22294745

[25] Taniguchi H He M Wu P Kim S Paik R Sugino K . A resource of Cre driver lines for genetic targeting of GABAergic neurons in cerebral cortex. Neuron. (2011) 71:995–1013. doi: 10.1016/j.neuron.2011.07.026, PMID: 21943598 PMC3779648

[26] Vong L Ye C Yang Z Choi B Chua S Jr. Lowell BB . Leptin action on GABAergic neurons prevents obesity and reduces inhibitory tone to POMC neurons. Neuron. (2011) 71:142–54. doi: 10.1016/j.neuron.2011.05.028, PMID: 21745644 PMC3134797

[27] Madisen L Zwingman TA Sunkin SM Oh SW Zariwala HA Gu H . A robust and high-throughput Cre reporting and characterization system for the whole mouse brain. Nat Neurosci. (2010) 13:133–40. doi: 10.1038/nn.2467, PMID: 20023653 PMC2840225

[28] Chen JS Salveridou E Liebmann L Sundaram SM Doycheva D Markova B . Triac treatment prevents neurodevelopmental and locomotor impairments in thyroid hormone transporter mct8/oatp1c1 deficient mice. Int J Mol Sci. (2023) 24. doi: 10.3390/ijms24043452, PMID: 36834863 PMC9966820

[29] Mayerl S Chen J Salveridou E Boelen A Darras VM Heuer H . Thyroid hormone transporter deficiency in mice impacts multiple stages of GABAergic interneuron development. Cereb Cortex. (2022) 32:329–41. doi: 10.1093/cercor/bhab211, PMID: 34339499 PMC8754375

[30] Racine RJ . Modification of seizure activity by electrical stimulation.2. Motor seizure. Electroencephalography Clin Neurophysiol. (1972) 32:281–&. doi: 10.1016/0013-4694(72)90177-0, PMID: 4110397

[31] De Vries EM Surovtseva O Vos WG Kunst RF Van Beeren M Kwakkel J . Downregulation of type 3 deiodinase in the hypothalamus during inflammation. Thyroid. (2019) 29:1336–43. doi: 10.1089/thy.2019.0201, PMID: 31303139

[32] Heuer H Schafer MK O’donnell D Walker P Bauer K . Expression of thyrotropin-releasing hormone receptor 2 (TRH-R2) in the central nervous system of rats. J Comp Neurol. (2000) 428:319–36. doi: 10.1002/1096-9861(20001211)428:2<319::AID-CNE10>3.0.CO;2-9 11064370

[33] Leger M Quiedeville A Bouet V Haelewyn B Boulouard M Schumann-Bard P . Object recognition test in mice. Nat Protoc. (2013) 8:2531–7. doi: 10.1038/nprot.2013.155, PMID: 24263092

[34] Mayerl S Alcaide Martin A Bauer R Schwaninger M Heuer H Ffrench-Constant C . Distinct actions of the thyroid hormone transporters mct8 and oatp1c1 in murine adult hippocampal neurogenesis. Cells. (2022) 11. doi: 10.3390/cells11030524, PMID: 35159334 PMC8834272

[35] Tremblay R Lee S Rudy B . GABAergic interneurons in the neocortex: from cellular properties to circuits. Neuron. (2016) 91:260–92. doi: 10.1016/j.neuron.2016.06.033, PMID: 27477017 PMC4980915

[36] Wilpert NM Krueger M Opitz R Sebinger D Paisdzior S Mages B . Spatiotemporal changes of cerebral monocarboxylate transporter 8 expression. Thyroid. (2020) 30:1366–83. doi: 10.1089/thy.2019.0544, PMID: 32143555

[37] Braun D Kinne A Brauer AU Sapin R Klein MO Köhrle J . Developmental and cell type-specific expression of thyroid hormone transporters in the mouse brain and in primary brain cells. Glia. (2011) 59:463–71. doi: 10.1002/glia.21116, PMID: 21264952

[38] Borgius L Restrepo CE Leao RN Saleh N Kiehn O . A transgenic mouse line for molecular genetic analysis of excitatory glutamatergic neurons. Mol Cell Neurosci. (2010) 45:245–57. doi: 10.1016/j.mcn.2010.06.016, PMID: 20600924

[39] Kawauchi D Ogg RJ Liu L Shih DJH Finkelstein D Murphy BL . Novel MYC-driven medulloblastoma models from multiple embryonic cerebellar cells. Oncogene. (2017) 36:5231–42. doi: 10.1038/onc.2017.110, PMID: 28504719 PMC5605674

[40] Le TN Zhou QP Cobos I Zhang S Zagozewski J Japoni S . GABAergic interneuron differentiation in the basal forebrain is mediated through direct regulation of glutamic acid decarboxylase isoforms by dlx homeobox transcription factors. J Neurosci. (2017) 37:8816–29. doi: 10.1523/JNEUROSCI.2125-16.2017, PMID: 28821666 PMC6596671

[41] Alevyzaki A Markova B De Angelis M Muller TD Chandrasekar A Muller-Fielitz H . Inactivation of thyroid hormone transporters mct8/oatp1c1 in mouse brain endothelial cells causes region-specific alterations in central thyroid hormone signaling. Thyroid. (2025) 35:816–27. doi: 10.1089/thy.2025.0089, PMID: 40622283

[42] Mayerl S Heuer H Ffrench-Constant C . Hippocampal neurogenesis requires cell-autonomous thyroid hormone signaling. Stem Cell Rep. (2020) 14:845–60. doi: 10.1016/j.stemcr.2020.03.014, PMID: 32302557 PMC7220957

[43] Kempermann G Song H Gage FH . Adult neurogenesis in the hippocampus. Hippocampus. (2023) 33:269–70. doi: 10.1002/hipo.23525, PMID: 36912499 PMC11175592

[44] Goncalves JT Schafer ST Gage FH . Adult neurogenesis in the hippocampus: from stem cells to behavior. Cell. (2016) 167:897–914. doi: 10.1016/j.cell.2016.10.021, PMID: 27814520

[45] Pelkey KA Chittajallu R Craig MT Tricoire L Wester JC Mcbain CJ . Hippocampal gabaergic inhibitory interneurons. Physiol Rev. (2017) 97:1619–747. doi: 10.1152/physrev.00007.2017, PMID: 28954853 PMC6151493

[46] Lim L Mi D Llorca A Marin O . Development and functional diversification of cortical interneurons. Neuron. (2018) 100:294–313. doi: 10.1016/j.neuron.2018.10.009, PMID: 30359598 PMC6290988

[47] Wonders CP Anderson SA . The origin and specification of cortical interneurons. Nat Rev Neurosci. (2006) 7:687–96. doi: 10.1038/nrn1954, PMID: 16883309

[48] Richard S Guyot R Rey-Millet M Prieux M Markossian S Aubert D . A pivotal genetic program controlled by thyroid hormone during the maturation of GABAergic neurons. iScience. (2020) 23:100899. doi: 10.1016/j.isci.2020.100899, PMID: 32092701 PMC7037980

[49] Jessberger S Clark RE Broadbent NJ Clemenson GD Jr. Consiglio A Lie DC . Dentate gyrus-specific knockdown of adult neurogenesis impairs spatial and object recognition memory in adult rats. Learn Mem. (2009) 16:147–54. doi: 10.1101/lm.1172609, PMID: 19181621 PMC2661246

[50] Revest JM Dupret D Koehl M Funk-Reiter C Grosjean N Piazza PV . Adult hippocampal neurogenesis is involved in anxiety-related behaviors. Mol Psychiatry. (2009) 14:959–67. doi: 10.1038/mp.2009.15, PMID: 19255582

[51] Richard S Aguilera N Thevenet M Dkhissi-Benyahya O Flamant F . Neuronal expression of a thyroid hormone receptor alpha mutation alters mouse behaviour. Behav Brain Res. (2017) 321:18–27. doi: 10.1016/j.bbr.2016.12.025, PMID: 28011173

[52] Maity-Kumar G Ständer L Deangelis M Lee S Molenaar A Becker L . Validation of dKO mice as a model organism for the Allan-Herndon-Dudley Syndrome. Mol Metab. (2022) 66. doi: 10.1016/j.molmet.2022.101616, PMID: 36270613 PMC9626936

[53] Navarro D Alvarado M Navarrete F Giner M Obregon MJ Manzanares J . Gestational and early postnatal hypothyroidism alters VGluT1 and VGAT bouton distribution in the neocortex and hippocampus, and behavior in rats. Front Neuroanat. (2015) 9:9. doi: 10.3389/fnana.2015.00009, PMID: 25741243 PMC4330898

[54] Zare Z Shafia S Mohammadi M . Thyroid hormone deficiency affects anxiety-related behaviors and expression of hippocampal glutamate transporters in male congenital hypothyroid rat offspring. Horm Behav. (2024) 162:105548. doi: 10.1016/j.yhbeh.2024.105548, PMID: 38636205

[55] Carobrez AP Bertoglio LJ . Ethological and temporal analyses of anxiety-like behavior: the elevated plus-maze model 20 years on. Neurosci Biobehav Rev. (2005) 29:1193–205. doi: 10.1016/j.neubiorev.2005.04.017, PMID: 16084592

[56] Liao XH Avalos P Shelest O Ofan R Shilo M Bresee C . AAV9-MCT8 delivery at juvenile stage ameliorates neurological and behavioral deficits in a mouse model of MCT8-deficiency. Thyroid. (2022) 32:849–59. doi: 10.1089/thy.2022.0034, PMID: 35350867 PMC9469747

[57] Nunez B Martinez De Mena R Obregon MJ Font-Llitjos M Nunes V Palacin M . Cerebral cortex hyperthyroidism of newborn mct8-deficient mice transiently suppressed by lat2 inactivation. PLoS One. (2014) 9:e96915. doi: 10.1371/journal.pone.0096915, PMID: 24819605 PMC4018440

[58] Friesema EC Jansen J Jachtenberg JW Visser WE Kester MH Visser TJ . Effective cellular uptake and efflux of thyroid hormone by human monocarboxylate transporter 10. Mol Endocrinol. (2008) 22:1357–69. doi: 10.1210/me.2007-0112, PMID: 18337592 PMC5419535

[59] Ceballos A Belinchon MM Sanchez-Mendoza E Grijota-Martinez C Dumitrescu AM Refetoff S . Importance of monocarboxylate transporter 8 for the blood-brain barrier-dependent availability of 3,5,3’-triiodo-L-thyronine. Endocrinology. (2009) 150:2491–6. doi: 10.1210/en.2008-1616, PMID: 19147674 PMC2671898

[60] Sundaram SM Arrulo Pereira A Muller-Fielitz H Kopke H De Angelis M Muller TD . Gene therapy targeting the blood-brain barrier improves neurological symptoms in a model of genetic MCT8 deficiency. Brain. (2022) 145:4264–74. doi: 10.1093/brain/awac243, PMID: 35929549 PMC9762946

