# Supplementary figures and images for: Neuron-specific expression of murine thyroid hormone transporters Mct8 and Oatp1c1 is dispensable for hippocampus-dependent neuronal functions
Source: Front Endocrinol (Lausanne). 2026 Mar 17;17:1781214. doi: 10.3389/fendo.2026.1781214 (PMC13035765; doi:10.3389/fendo.2026.1781214)

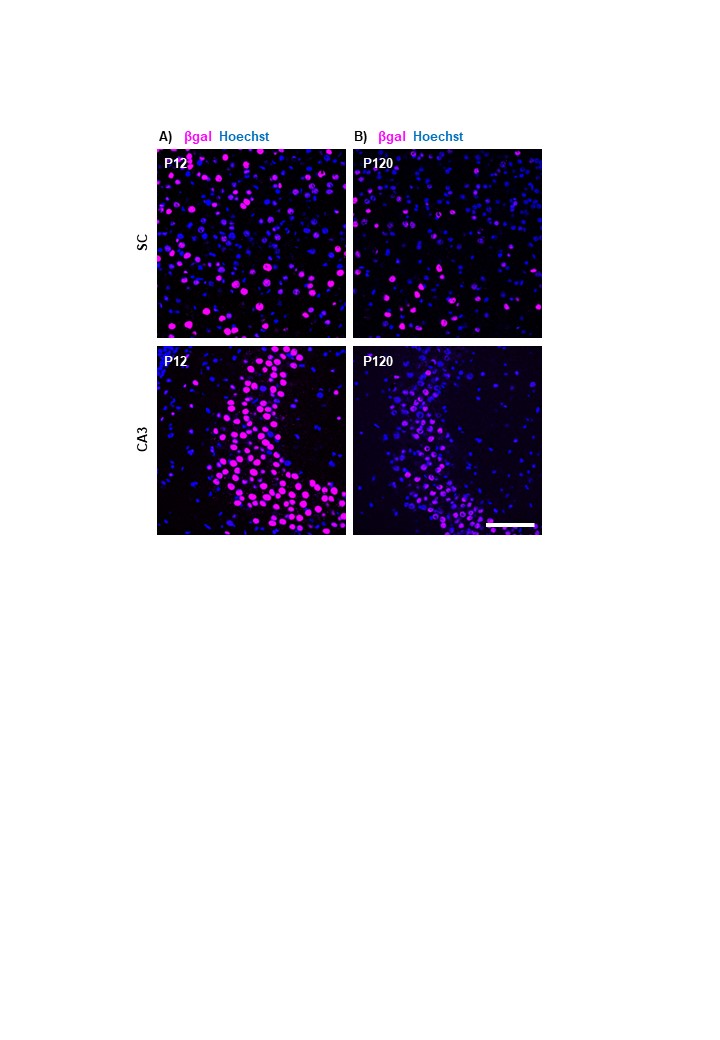

Supplement: Supplementary Figure 1 — βgal expression in Mct8-KO animals. Perfusion-fixed coronal brain slices from Mct8-KO animals were subjected to immunofluorescence analysis. Representative pictures of βgal staining (magenta) and Hoechst 33258-stained nuclei (blue) in the somatosensory cortex (SC) and CA3 area of the hippocampus of mice at P12 (A) and P120 (B). Scale bar: 100 µm. [file Image1.jpeg]

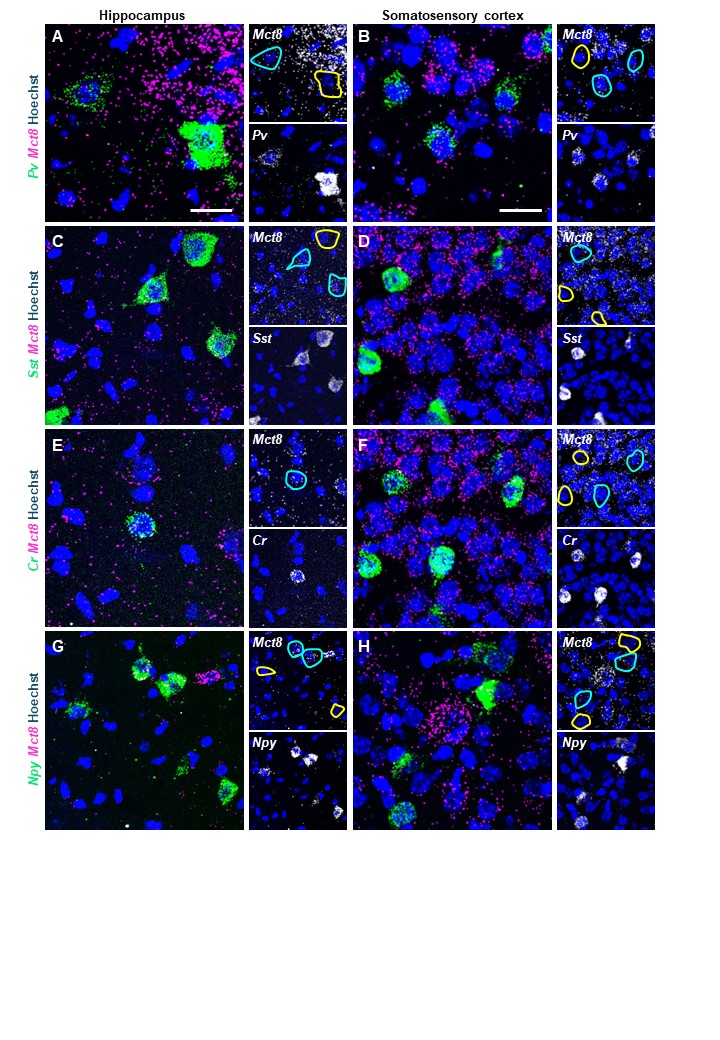

Supplement: Supplementary Figure 2 — Co-expression of Mct8 mRNA with GABAergic markers in wild-type hippocampus at P12. Fresh frozen coronal brain slices were subjected to FISH. Fluorescent images showing Mct8 mRNA (in magenta) co-localization with Pv(A, B), Sst(C, D), Cr(E, F) and Npy(G, H) (all in green) in the hippocampus (A, C, E, G) and somatosensory cortex (B, D, F, H). Single Mct8 and GABAergic marker channels are presented in grey scale. Hoechst 33258-stained nuclei are shown in blue in all images. Cyan circles indicate co-localization of Mct8 with GABAergic marker while yellow circles indicate absence of Mct8 expression. n = 4. Scale bar: 20 µm. [file Image2.jpeg]

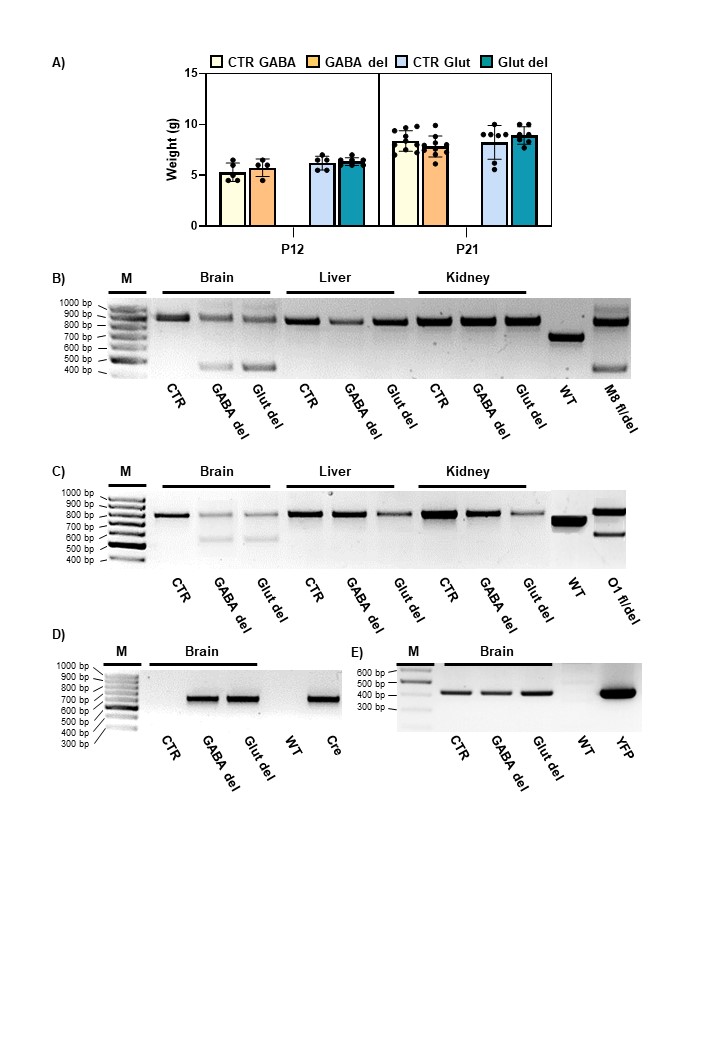

Supplement: Supplementary Figure 3 — Evaluation of conditional animals. The body weight of the animals was recorded at postnatal days P12 and P21 (A). Genomic DNA derived from liver, kidney and brain homogenates of control (CTR), GABA del and Glut del mice at P120 was subjected to PCR analysis. Detection of Mct8 (M8) (B) and Oatp1c1 (O1) (C) flox (fl) and del alleles in different tissues. Confirmation of Cre (D) and YFP (E) transgene presence in brains of the different experimental animals. M, molecular-sized weight marker in base pairs (bp); WT, wild-type allele. [file Image3.jpeg]

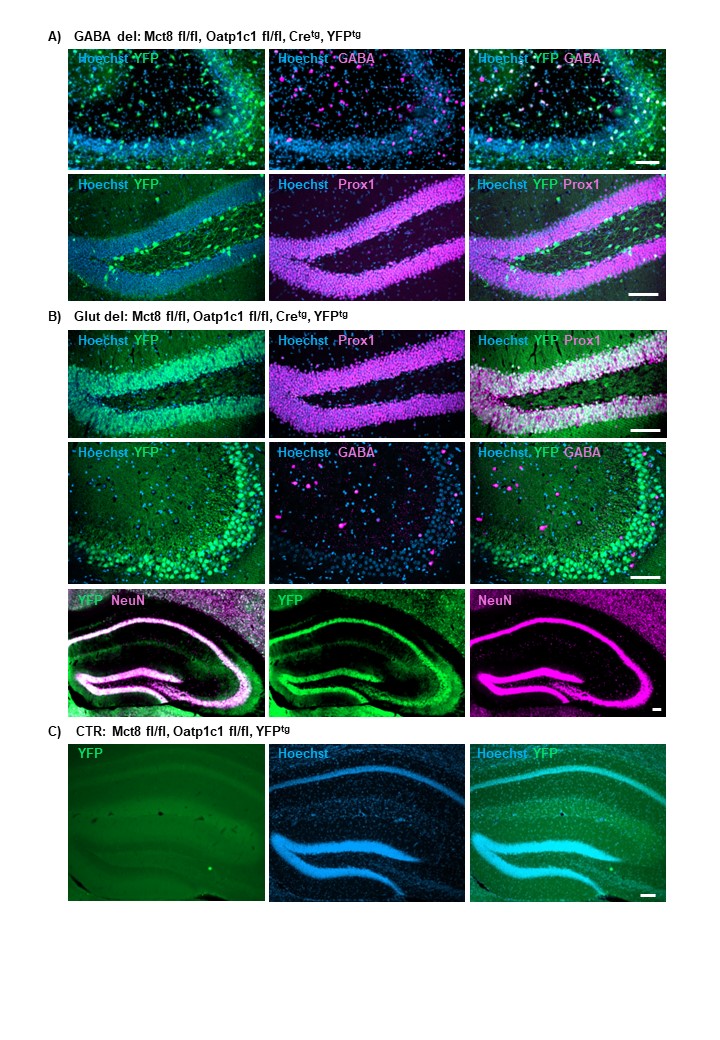

Supplement: Supplementary Figure 4 — YFP immunostaining in hippocampal sections of GABA del and Glut del mice at P120. Representative immunohistochemical images of coronal vibratome sections depicting YFP (green) as an indicator of Cre recombination. Co-staining with antibodies against GABA, Prox1, or NeuN (all shown in magenta) was performed to visualize GABAergic, glutamatergic, or general neuronal populations, respectively, in the hippocampal CA3 or DG regions in GABA del (A) and Glut del mice (B). Representative immunofluorescence images of YFP in control (CTR) mice with no Cre activity (C). Hoechst33258-stained nuclei are shown in blue in all images. Scale bar: 100 µm. [file Image4.jpeg]
